# Sex-Specific Differences in Hemodialysis Prevalence and Practices and the Male-to-Female Mortality Rate: The Dialysis Outcomes and Practice Patterns Study (DOPPS)

**DOI:** 10.1371/journal.pmed.1001750

**Published:** 2014-10-28

**Authors:** Manfred Hecking, Brian A. Bieber, Jean Ethier, Alexandra Kautzky-Willer, Gere Sunder-Plassmann, Marcus D. Säemann, Sylvia P. B. Ramirez, Brenda W. Gillespie, Ronald L. Pisoni, Bruce M. Robinson, Friedrich K. Port

**Affiliations:** 1 Clinical Division of Nephrology & Dialysis, Department of Internal Medicine III, Medical University of Vienna, Vienna, Austria; 2 Arbor Research Collaborative for Health, Ann Arbor, Michigan, United States of America; 3 Centre Hospitalier de l'Université de Montréal, Montreal, Quebec, Canada; 4 Gender Medicine Unit, Clinical Division of Endocrinology & Metabolism, Department of Internal Medicine III, Medical University of Vienna, Vienna, Austria; 5 Department of Biostatistics, University of Michigan, Ann Arbor, Michigan, United States of America; Royal Derby Hospital, United Kingdom

## Abstract

**Background:**

A comprehensive analysis of sex-specific differences in the characteristics, treatment, and outcomes of individuals with end-stage renal disease undergoing dialysis might reveal treatment inequalities and targets to improve sex-specific patient care. Here we describe hemodialysis prevalence and patient characteristics by sex, compare the adult male-to-female mortality rate with data from the general population, and evaluate sex interactions with mortality.

**Methods and Findings:**

We assessed the Human Mortality Database and 206,374 patients receiving hemodialysis from 12 countries (Australia, Belgium, Canada, France, Germany, Italy, Japan, New Zealand, Spain, Sweden, the UK, and the US) participating in the international, prospective Dialysis Outcomes and Practice Patterns Study (DOPPS) between June 1996 and March 2012. Among 35,964 sampled DOPPS patients with full data collection, we studied patient characteristics (descriptively) and mortality (via Cox regression) by sex. In all age groups, more men than women were on hemodialysis (59% versus 41% overall), with large differences observed between countries. The average estimated glomerular filtration rate at hemodialysis initiation was higher in men than women. The male-to-female mortality rate ratio in the general population varied from 1.5 to 2.6 for age groups <75 y, but in hemodialysis patients was close to one. Compared to women, men were younger (mean = 61.9±standard deviation 14.6 versus 63.1±14.5 y), were less frequently obese, were more frequently married and recipients of a kidney transplant, more frequently had coronary artery disease, and were less frequently depressed. Interaction analyses showed that the mortality risk associated with several comorbidities and hemodialysis catheter use was lower for men (hazard ratio [HR] = 1.11) than women (HR = 1.33, interaction *p*<0.001). This study is limited by its inability to establish causality for the observed sex-specific differences and does not provide information about patients not treated with dialysis or dying prior to a planned start of dialysis.

**Conclusions:**

Women's survival advantage was markedly diminished in hemodialysis patients. The finding that fewer women than men were being treated with dialysis for end-stage renal disease merits detailed further study, as the large discrepancies in sex-specific hemodialysis prevalence by country and age group are likely explained by factors beyond biology. Modifiable variables, such as catheter use, showing significant sex interactions suggest interventional targeting.

*Please see later in the article for the Editors' Summary*

## Abstract 2

In this study, Port and colleagues describe hemodialysis prevalence and patient characteristics by sex, compare men-to-women mortality rate with data from the general population, and evaluate sex interactions with mortality. The results show that women's survival advantage was markedly diminished in hemodialysis patients.

*Please see later in the article for the Editors' Summary*

## Editors' Summary

**Background:**

Throughout life, the kidneys filter waste products (from the normal breakdown of tissues and from food) and excess water from the blood to make urine. Chronic kidney disease—an increasingly common condition globally—gradually destroys the kidney's filtration units (the nephrons). As the nephrons stop working, the rate at which the blood is filtered (the glomerular filtration rate) decreases, and waste products build up in the blood, eventually leading to life-threatening end-stage kidney (renal) disease. Symptoms of chronic kidney disease, which rarely occur until the disease is advanced, include tiredness, swollen feet and ankles, and frequent urination, particularly at night. Chronic kidney disease cannot be cured, but its progression can be slowed by controlling diabetes and other conditions that contribute to its development. End-stage kidney disease is treated by regular hemodialysis (a process in which blood is cleaned by passing it through a filtration machine) or by kidney transplantation.

**Why Was This Study Done?:**

Like many other long-term conditions, the prevalence (the proportion of the population that has a specific disease) of chronic kidney disease and of end-stage renal disease, and treatment outcomes for these conditions, may differ between men and women. Some of these sex-specific differences may arise because of sex-specific differences in normal biological functions. Other sex-specific differences may be related to sex-specific differences in patient care or in patient awareness of chronic kidney disease. A comprehensive analysis of sex-specific differences among individuals with end-stage renal disease might identify both treatment inequalities and ways to improve sex-specific care. Here, in the Dialysis Outcomes and Practice Patterns Study (DOPPS), the researchers investigate sex-specific differences in the prevalence and practices of hemodialysis and in the characteristics of patients undergoing hemodialysis, and investigate the adult male-to-female mortality (death) rate among patients undergoing hemodialysis. The DOPPS is a prospective cohort study that is investigating the characteristics, treatment, and outcomes of adult patients undergoing hemodialysis in representative facilities in 19 countries (12 countries were available for analysis at the time of the current study).

**What Did the Researchers Do and Find?:**

To investigate sex-specific differences in hemodialysis prevalence, the researchers compared data from the Human Mortality Database, which provides detailed population and mortality data for 37 countries, with data collected by the DOPPS. Forty-one percent of DOPPS patients were women, compared to 52% of the general population in 12 of the DOPPS countries. Next, the researchers used data collected from a randomly selected subgroup of patients to examine sex-specific differences in patient characteristics and mortality. The average estimated glomerular filtration rate at hemodialysis initiation was higher in men than women. Moreover, men were more frequently recipients of a kidney transplant than women. Notably, although in the general population in a given age group women were less likely to die than men, among hemodialysis patients, women were as likely to die as men. Finally, the researchers investigated which patient characteristics were associated with the largest sex-specific differences in mortality risk. The use of a hemodialysis catheter (a tube that is inserted into a patient's vein to transfer their blood into the hemodialysis machine) was associated with a lower mortality risk in men than in women.

**What Do These Findings Mean?:**

These findings show that, among patients treated with hemodialysis for end-stage renal disease, women differ from men in many ways. Although some of these sex-specific differences may be related to biology, others may be related to patient care and to patient awareness of chronic kidney disease. Because this is an observational study, these findings cannot prove that the reported differences in hemodialysis prevalence, treatment, and mortality are actually caused by being a man or a woman. Importantly, however, these findings suggest that hemodialysis may abolish the survival advantage that women have over men in the general population and that fewer women than men are being treated for end-stage-renal disease, even though chronic kidney disease is more common in women than in men. Finally, the finding that the use of hemodialysis catheters for access to veins is associated with a higher mortality risk among women than among men suggests that, where possible, women should be offered a surgical process called arteriovenous fistula placement, which is recommended for access to veins during long-term hemodialysis but which may, in the past, have been underused in women.

**Additional Information:**

Please access these websites via the online version of this summary at http://dx.doi.org/10.1371/journal.pmed.1001750.

- More information about the DOPPS program is available
- The US National Kidney and Urologic Diseases Information Clearinghouse provides information about all aspects of kidney disease; the US National Kidney Disease Education Program provides resources to help improve the understanding, detection, and management of kidney disease (in English and Spanish)
- The UK National Health Service Choices website provides information for patients on chronic kidney disease and about hemodialysis, including some personal stories
- The US National Kidney Foundation, a not-for-profit organization, provides information about chronic kidney disease and about hemodialysis (in English and Spanish)
- The not-for-profit UK National Kidney Federation provides support and information for patients with kidney disease and for their carers, including information and personal stories about hemodialysis
- World Kidney Day, a joint initiative between the International Society of Nephrology and the International Federation of Kidney Foundations, aims to raise awareness about kidneys and kidney disease
- MedlinePlus has pages about chronic kidney disease and about hemodialysis

## Introduction

Because differences in men's and women's physiology have widely been recognized [1], researchers are encouraged to evaluate clinical study data by sex [2],[3]. Important sex-specific distinctions have been recognized in several of the most prevalent medical conditions, such as obesity [4], type 2 diabetes mellitus [5],[6], cardiovascular disease [7],[8], and depression [9]. Many of these conditions coexist with, or may have contributed to, chronic kidney disease [10]. Chronic kidney disease in itself raises numerous gender questions, for example, regarding sex-dependent prevalence [11] and disease awareness [12]. Sex-specific differences in the characteristics, treatment, and outcomes for individuals on renal replacement therapy have, however, only once previously been the primary theme in an international study, and with focus on mortality patterns at the start of dialysis [13].

Here we present a large adult male-to-female comparison of patient and treatment characteristics as well as mortality risk, using evidence from participants in the international Dialysis Outcomes and Practice Patterns Study (DOPPS). We also compare the adult male-to-female mortality risk with that of the general population, as deduced from the Human Mortality Database life tables. We aimed to describe current hemodialysis practice patterns, and identify patient variables or hemodialysis practices that can be modified in order to improve the care of women and men with end-stage renal disease by assessing (1) hemodialysis prevalence among study participants, overall and by country, (2) national differences in sex-dependent hemodialysis patient mortality, (3) sex-dependent differences in hemodialysis characteristics, and (4) the presence of a sex interaction in the associations between hemodialysis characteristics and mortality.

## Methods

### Patients and Data Collection

#### DOPPS data

The DOPPS is an international prospective cohort study of adult patients (ages ≥18 y) undergoing hemodialysis treated in representative facilities of each participating country (Australia, Belgium, Canada, France, Germany, Italy, Japan, New Zealand, Spain, Sweden, the United Kingdom, and the United States). Phase 1 of the DOPPS collected data from June 1996 to October 2001, Phase 2 from February 2002 to February 2005, Phase 3 from June 2005 to January 2009, and Phase 4 from March 2009 to March 2012. Data collection in Australia, Belgium, Canada, New Zealand, and Sweden did not begin until Phase 2. Due to the small number of DOPPS facilities recruited in New Zealand (*n* = 2), patients in this country were combined with those in Australia (*n* = 18 facilities) in subsequent analyses. DOPPS facilities were enrolled randomly from a list of all hemodialysis facilities within each nation at the beginning of each phase of data collection between 1996 and 2012, as described previously [14],[15]. In the current study, we analyzed the following patient populations: (1) 206,374 DOPPS census patients from the initial cross-section of patients in each study phase, i.e., all patients dialyzing in the DOPPS facilities at study start, having data on demographics and mortality; (2) 35,964 prevalent patients (subset of patient population #1 above, based on a random selection of 20–40 hemodialysis patients per participating facility); and (3) 14,941 incident patients from patient population #1 above who were enrolled in the DOPPS within 90 d after initiation of hemodialysis therapy between March 2009 and March 2012.

Study approval was received annually from a central institutional review board. Additional national and local ethics committee approvals and written patient consents were obtained as required. Demographic data (including race), comorbid conditions, laboratory values, and medications for sampled patients were abstracted from patient records. Mortality events were collected during study follow-up. Estimated glomerular filtration rate (eGFR) at dialysis initiation was calculated among a subset of population #3 (described above) using the Modification of Diet in Renal Disease Study (MDRD) formula [16].

#### The Human Mortality Database

To compare mortality rates for the general population with those of the DOPPS population, data from the Human Mortality Database was used [17]. Country- and age-group-specific mortality rates were calculated using data from January 2000–December 2009. Individuals aged <18 y were excluded. Dates of deaths were obtained from national registries, and population size was determined from census data (for the year of the census) and inter-censual calculations (for years between censuses). Time at risk for each age group was corrected for the timing of deaths during the interval. Additional details are available in the Human Mortality Database methods protocol ([18], pg. 7–10).

#### Data from national hemodialysis registries in the DOPPS countries

For comparisons with DOPPS results, we used data from national hemodialysis registries in Australia/New Zealand [19]; Canada [20]; Japan [21]; Belgium, France, Spain, Sweden, and the United Kingdom [22]; and the United States [23].

### Data Analysis

**Figure 1:**
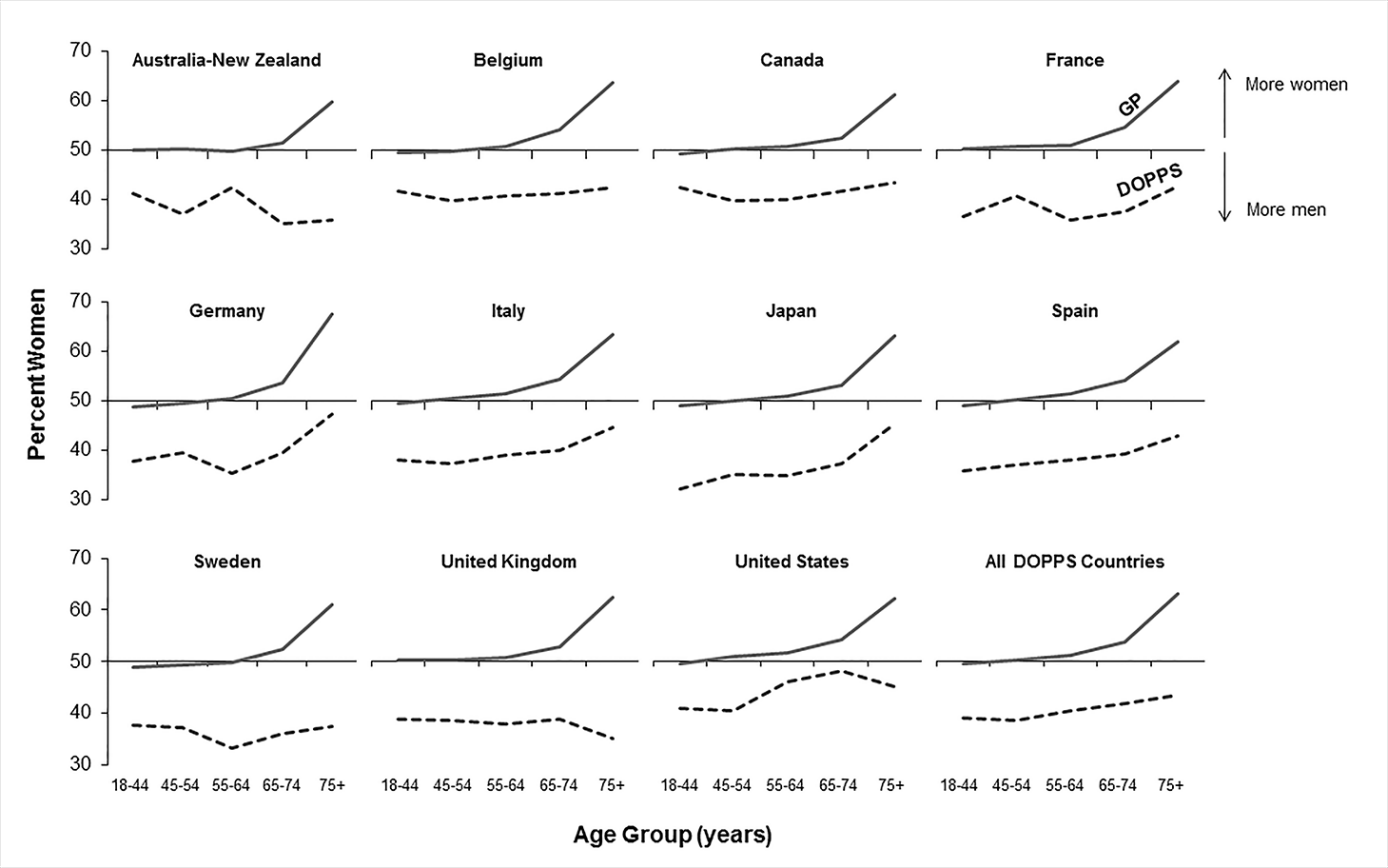
Percent of population that are women, by age group, in the hemodialysis and general populations. DOPPS data are prevalent hemodialysis patients from the DOPPS census (1996–2012). General population (GP) data are from the Human Mortality Database between 2000 and 2009.

**Figure 2:**
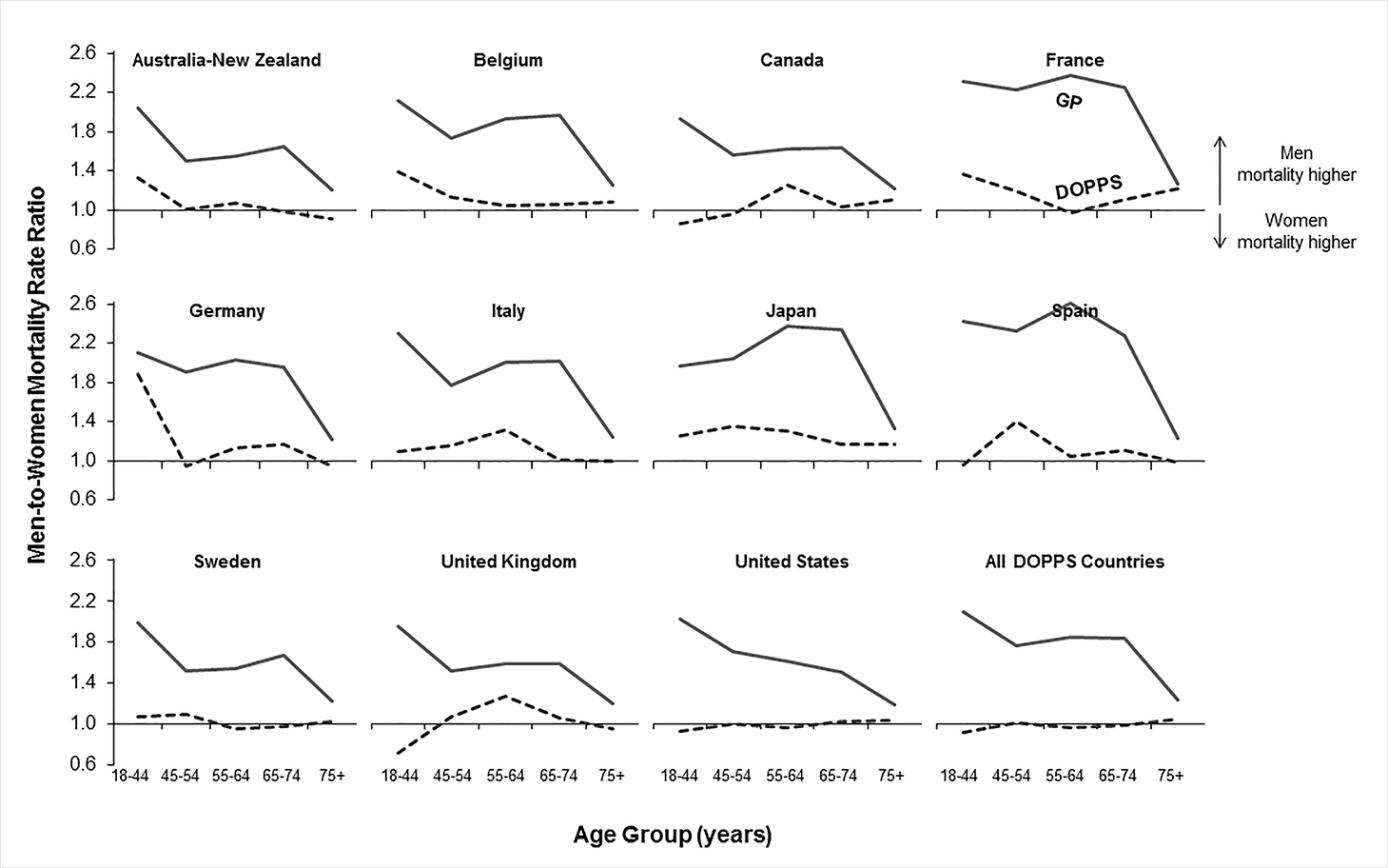
Adult male-to-female mortality rate ratio, by age group, in the hemodialysis and general populations. Mortality rate ratios are unadjusted. DOPPS data are prevalent hemodialysis patients from the DOPPS census (1996–2012). General population (GP) data are from the Human Mortality Database between 2000 and 2009.

**Figure 3:**
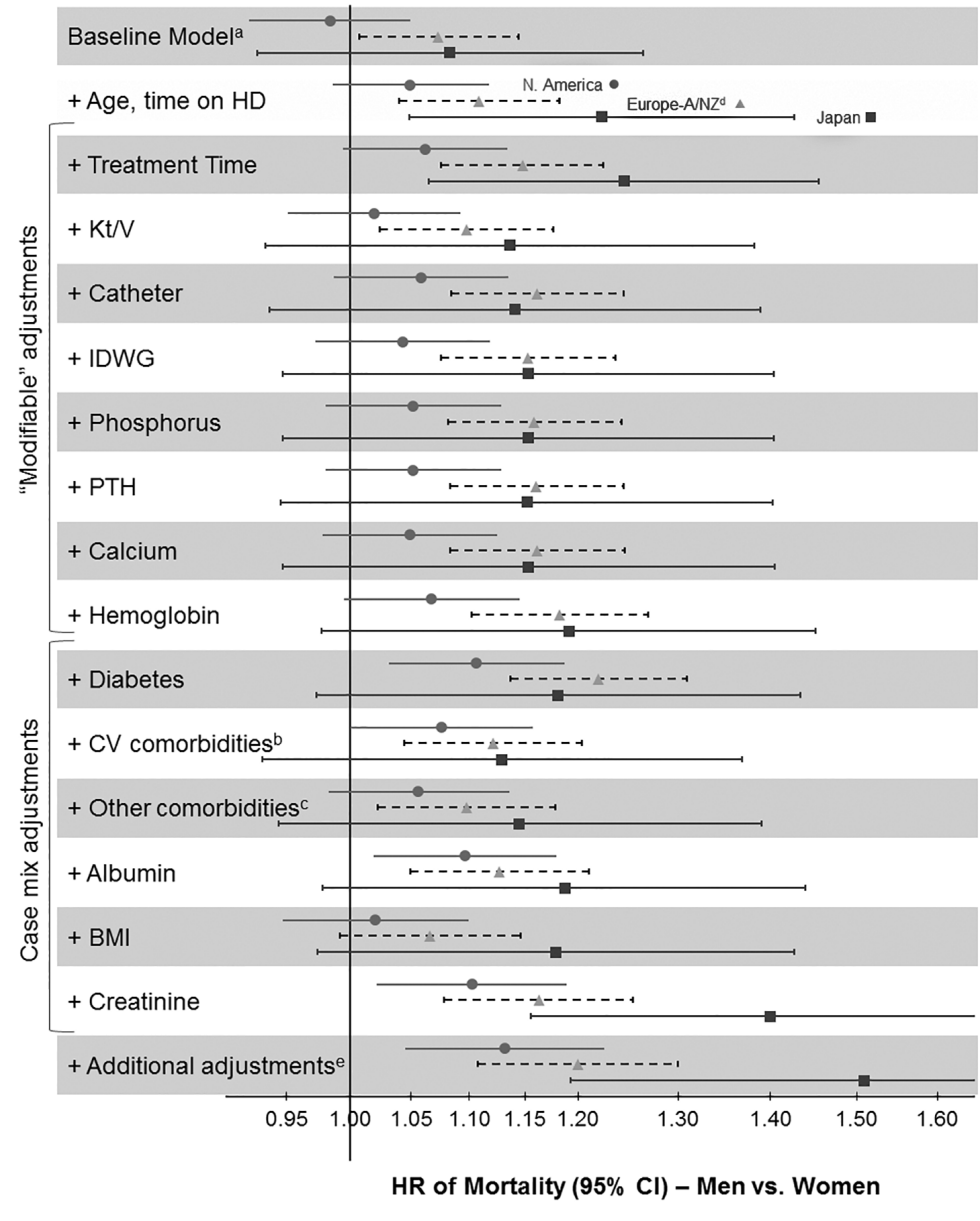
Adjusted hazard ratios for the adult male-to-female mortality risk in hemodialysis patients, by region. ^a^Stratified by country (including US black race and US non-black race) and phase; *n* = 36,216 patients (*n* = 8,258 deaths) among patients with time on dialysis >90 d dialyzing 3× weekly. ^b^Coronary artery disease, cerebrovascular disease, congestive heart failure, hypertension, peripheral vascular disease, other cardiovascular disease. ^c^Cancer, gastrointestinal bleed, lung disease, neurologic disorder, psychologic disorder, recurrent cellulitis. ^d^European countries = Belgium, France, Germany, Italy, Spain, Sweden, UK. ^e^Education, employment, marital status, smoking status, predialysis systolic blood pressure, blood flow rate, serum potassium, medication prescriptions (erythopoiesis-stimulating agent, phosphate binder, vitamin D, antihypertensive, antibiotic), prior parathyroidectomy, and prior transplant. A/NZ, Australia/New Zealand; BMI, body mass index; CV, cardiovascular; HD, hemodialysis; IDWG, interdialytic weight gain; N. America, North America; PTH, parathyroid hormone.

**Figure 4:**
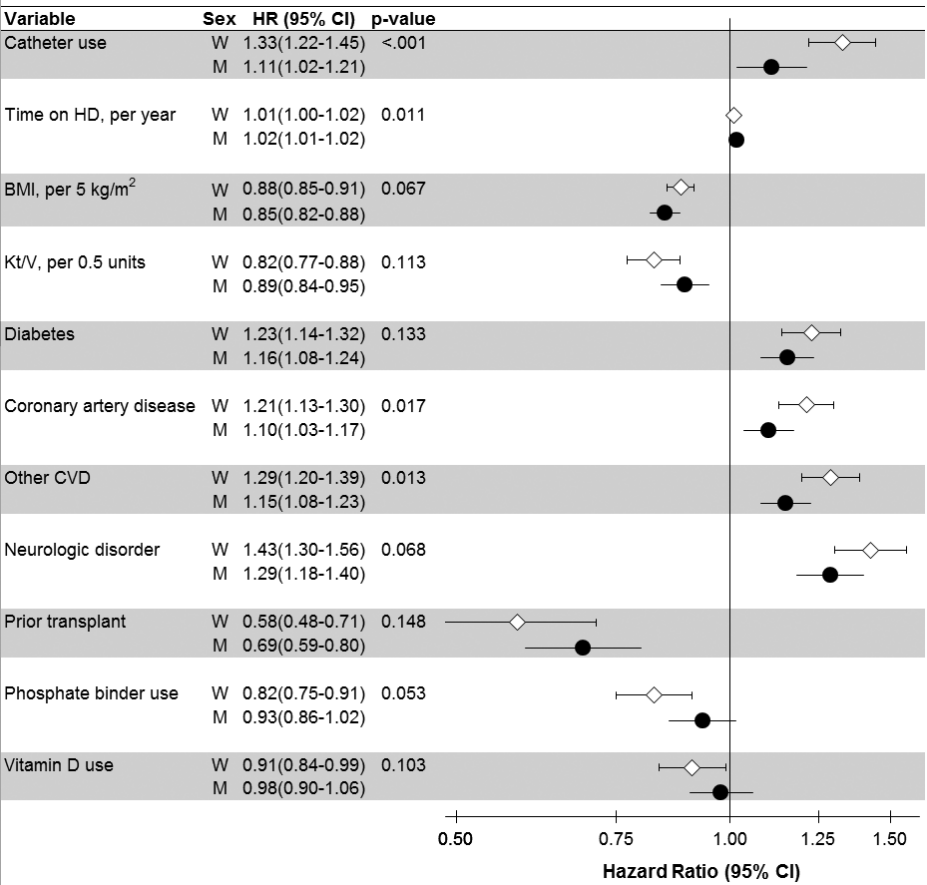
Analysis of sex interaction in the associations between hemodialysis patient characteristics and mortality. *p*-Value is for interaction with sex, shown for variables with *p*<0.15 (*n* = 37 interactions tested). Adjusted for all variables listed in Tables 2 and 3, in addition to variables listed in Figure 3. BMI, body mass index; CI, confidence interval; CVD, cardiovascular disease; HD, hemodialysis; M, men; W, women.

Age groups for the male-to-female mortality rate ratios in Figures 1 and 2 were chosen based on the DOPPS sample protocol (≥18 y) and the average age of patients on hemodialysis. The primary outcome of interest in the current study was mortality, and the primary exposure of interest was patient sex. Variables adjusted for as potential confounders are listed in Figure 3, including age, time on dialysis, and numerous other patient and treatment characteristics. The same variables were also assessed as potential effect modifiers in Figure 4. Standard descriptive statistics were used to characterize the DOPPS patients included in the study.

#### Cox regression

Cox regression was used for DOPPS patients from date of entry into DOPPS phases (Phases 1–4) to analyze the association between patient sex and mortality, stratified by country and phase, accounting for facility clustering using robust sandwich covariance estimators, and adjusted for the variables listed in Figure 3. Time at risk started at study enrollment and ended at the time of death, time of kidney transplantation, 7 d after leaving the facility because of transfer, 7 d after changing modality, time of loss to follow-up, or end of the study phase. The median follow-up time was 1.7 y, and the percentages of censoring or outcome events ordered by frequency were study end (57%), death (22%), facility transfer (12%), kidney transplantation (6%), modality change (1%), and other and loss to follow-up (<1%). The proportional hazards assumption was confirmed by testing interactions between covariates and time at risk and plotting log-log survival curves versus time.

#### Multiple imputation

Overall, the rate of missing data for covariates was low (e.g., <2% for the majority of covariates; up to 25% for one variable included in the models). For missing data, we used the Sequential Regression Multiple Imputation Method implemented by IVEware [24]. All analyses were performed using SAS software, version 9.2 (SAS Institute, Cary, North Carolina). The authors have followed the suggestions of the STROBE Statement guidelines for reporting observational studies [25].

## Results

### Prevalence of Hemodialysis Treatment for End-Stage Renal Disease by Sex

According to the Human Mortality Database, 315,950,449 men and 336,229,337 women adults were alive in the year 2009 in the evaluated counties, equivalent to a proportion of 52% women. Of all 206,374 DOPPS hemodialysis patients included in the cross-sectional census analysis, 121,566 were men and 84,808 were women, equivalent to a proportion of 41% women. The finding that fewer women than men were on hemodialysis in the DOPPS, although more women than men were alive in the general population, was consistent even for incident dialysis patients throughout the five age groups we analyzed (Table 1), indicating that differences in early dialysis mortality had not influenced the results observed in the prevalent cross-section. For individuals aged ≥75 y, along with a pronounced increase in the proportion of women compared to men in the general population, the proportion of women compared to men on hemodialysis increased in seven of the 12 countries, but remained below 50% (Figure 1). Throughout age groups, we observed large national differences in the proportion of women compared to men on hemodialysis. By age group, the highest proportion of women compared to men on hemodialysis was observed in the United States in the age group 65–74 y (49.2%), while the lowest proportion of women compared to men on hemodialysis was observed in Australia/New Zealand in the age group ≥75 y (31.9%). The findings shown in Figure 1 were confirmed by analyzing data from national hemodialysis registries in ten of the DOPPS countries with publicly available registry data (Table S1). The average eGFR (by MDRD formula) at the start of dialysis was 10.6 ml/min/1.73 m^2^ in male incident patients and 10.1 ml/min/1.73 m^2^ in female incident patients in DOPPS Phase 4, suggesting that dialysis was initiated later in women in the course of end-stage renal disease. This relationship was consistent in all DOPPS countries.

**Table 1:** Percentage of the population that are women in the hemodialysis versus general population by age group (2009).

Country	Age Group														
	18–44 y			45–54 y			55–64 y			65–74 y			75+ y		
	GP	HD_p_	HD_i_	GP	HD_p_	HD_i_	GP	HD_p_	HD_i_	GP	HD_p_	HD_i_	GP	HD_p_	HD_i_
Australia-New Zealand	49.8	49.1	25.3	50.6	42.3	27.7	50.3	43.8	41.6	51.2	42.4	30.6	58.4	31.9	24.6
Belgium	49.6	42.5	41.0	49.7	34.0	39.8	50.4	42.1	37.6	53.4	43.8	38.1	62.6	42.1	39.7
Canada	49.4	43.9	38.0	50.0	40.8	33.5	50.8	42.9	38.9	52.2	39.3	38.4	60.0	48.9	40.1
France	50.2	35.6	33.7	51.1	40.1	37.2	51.3	36.2	38.4	53.9	39.3	33.1	63.3	45.9	37.5
Germany	49.1	39.4	32.4	49.4	34.9	47.1	50.6	39.8	32.7	52.9	35.0	31.3	64.4	45.9	41.5
Italy	49.5	35.6	37.3	50.6	36.3	37.1	51.4	38.5	36.3	53.7	38.8	32.9	62.6	45.5	37.4
Japan	49.1	29.4	32.4	49.9	32.1	27.5	50.8	35.8	28.6	52.9	37.7	30.0	62.3	43.6	38.8
Spain	48.6	34.4	37.2	50.2	37.4	32.8	51.5	36.3	31.0	53.8	40.0	27.4	61.2	42.9	37.8
Sweden	48.9	44.4	27.0	49.3	42.6	35.6	49.9	34.9	26.0	51.4	32.9	32.5	60.4	40.0	35.1
United Kingdom	50.1	40.9	41.7	50.5	40.1	26.9	50.8	39.2	42.2	52.3	45.0	37.7	60.7	40.2	36.1
United States	49.7	41.5	41.6	50.8	39.1	42.5	51.8	44.9	43.3	53.6	49.2	39.3	61.1	46.3	40.8

General population (GP) data are from the Human Mortality Database, 2009. Hemodialysis prevalent population (HD_p_) data are from the initial census of patients in facilities at the start of DOPPS Phase 4 (2009), cross-sectional, regardless of duration of end-stage renal disease. Hemodialysis incident population (HD_i_) restricted to patients on dialysis <90 d when added to the facility census of patients in the DOPPS (includes patients in DOPPS facilities at the start of DOPPS Phase 4 data collection as well as patients who started dialyzing at the DOPPS facility during 2009–2012).

GP, general population; HD_p_, hemodialysis prevalent population; HD_i_, hemodialysis incident population.

### Male-to-Female Mortality Ratio during Hemodialysis and in the General Population

We calculated crude adult male-to-female mortality rate ratios for the general populations of the countries participating in the DOPPS using the Human Mortality Database life tables. Within all of the five age groups analyzed, men's mortality surpassed women's mortality, with male-to-female mortality rate ratios above two observed in France, Spain, and Japan (Figure 2). In the census of the DOPPS hemodialysis population, however, men's and women's mortality were very similar, with male-to-female mortality rate ratios very close to one throughout the five age groups in all DOPPS countries except Japan. These data indicate that the survival advantage that women have over men in the general population was markedly diminished in hemodialysis patients with end-stage renal disease, consistent with previous findings in incident dialysis patients [13]. The male-to-female mortality rate ratio varied by age group and country, particularly in the lowest age group (18–44 y), where men on hemodialysis from Australia/New Zealand, Belgium, France, Germany, and Japan had—compared to women—a higher risk of mortality, while it was overall very close to equality in other countries (Figure 2).

### Characteristics of the Hemodialysis Population, by Sex and Region

Numerous patient and treatment characteristics differed significantly between all men and women on hemodialysis in the DOPPS sample (Table 2). Among demographics, patient age and time on hemodialysis differed slightly, with women on average being older (by 1.2 y) and having longer time on dialysis (by 0.3 y) than men. Large differences between men and women, however, were observed for marital status (more men than women married), employment (more men than women employed), smoking (more men than women smokers), and level of education (more women than men with education less than high school). Women also had higher body mass index and, accordingly, were more frequently obese (19.2% women versus 11.9% men with body mass index ≥30 kg/m^2^). These demographic trends were largely consistent across regions, with the exception of time on dialysis in North America (similar for men and women) and body mass index in Japan (slightly higher in men than in women).

**Table 2:** Patient characteristics, by sex and region.

Characteristic	All Patients		Patients By Region					
			Europe and Australia/New Zealand		North America		Japan	
	Men (*n* = 17,109)	Women (*n* = 12,733)	Men (*n* = 7,836)	Women (*n* = 5,647)	Men (*n* = 5,190)	Women (*n* = 4,453)	Men (*n* = 4,083)	Women (*n* = 2,633)
**Demographics**								
Age, years	**61.9 (14.6)**	**63.1 (14.5)**	**63.4 (14.9)**	**64.0 (14.8)**	**60.6 (15.5)**	**62.5 (15.1)**	**60.8 (12.5)**	**62.2 (12.7)**
Time on hemodialysis, years	**5.3 (5.7)**	**5.6 (5.8)**	**4.9 (5.5)**	**5.4 (5.9)**	3.9 (4.1)	3.9 (4.1)	**7.8 (6.9)**	**8.7 (6.9)**
Body mass index, kg/m^2^	**24.4 (5.0)**	**24.9 (6.5)**	**24.9 (4.5)**	**25.3 (5.9)**	**26.0 (5.8)**	**27.4 (7.2)**	**21.2 (2.9)**	**20.1 (3.2)**
Body mass index ≥30 kg/m^2^	**11.9**	**19.2**	**12.1**	**18.5**	**20.5**	**31.3**	0.9	0.8
Black race, percent	**10.2**	**12.8**	1.9	1.9	**30.7**	**34.2**	-	-
Married, percent	**65.6**	**48.6**	**66.5**	**49.5**	**55.8**	**37.3**	**76.1**	**65.3**
Employed, percent	**20.7**	**8.0**	**13.8**	**6.9**	**12.4**	**6.8**	**45.2**	**12.6**
Smoker, percent	**21.6**	**9.4**	**17.1**	**8.9**	**20.7**	**12.2**	**32.1**	**6.3**
Education less than high school, percent	**45.5**	**54.4**	**59.9**	**72.1**	**35.6**	**40.5**	**29.3**	**37.6**
**Laboratory values**								
S. phosphorus, mmol/l	1.8 (0.6)	1.8 (0.6)	**1.7 (0.6)**	**1.7 (0.6)**	1.8 (0.6)	1.8 (0.6)	**1.8 (0.5)**	**1.8 (0.5)**
S. calcium, mmol/l	**2.3 (0.2)**	**2.3 (0.2)**	**2.3 (0.2)**	**2.3 (0.3)**	**2.3 (0.2)**	**2.3 (0.2)**	**2.3 (0.2)**	**2.3 (0.2)**
S. PTH, ng/l	**272 (301)**	**289 (324)**	**277 (295)**	**304 (335)**	321 (348)	317 (347)	189 (212)	198 (219)
S. potassium, mmol/l	**5.0 (0.8)**	**5.0 (0.8)**	5.2 (0.8)	5.2 (0.8)	**4.8 (0.7)**	**4.9 (0.7)**	5.1 (0.8)	5.0 (0.8)
S. sodium, mmol/l	**138.4 (3.5)**	**138.0 (3.6)**	**138.2 (3.6)**	**137.7 (3.7)**	**138.1 (3.5)**	**137.8 (3.5)**	139.1 (3.2)	139.0 (3.2)
S. albumin, g/l	**38.1 (4.8)**	**37.3 (4.6)**	**38.1 (5.3)**	**37.4 (5.2)**	**37.8 (4.4)**	**36.8 (4.3)**	**38.2 (4.2)**	**37.7 (3.9)**
Hemoglobin, g/l	**112 (16)**	**111 (15)**	**116 (15)**	**114 (15)**	**115 (15)**	**113 (14)**	**103 (13)**	**99.4 (12.7)**
C-reactive protein, nmol/l^a^	**117 (215)**	**105 (205)**	**150 (238)**	**135 (226)**	—	—	50.2 (139.3)	42.1 (129.4)
Uric acid, µmol/l	**409 (93)**	**394 (89)**	**388 (88)**	**372 (82)**	**387 (91)**	**366 (81)**	453 (85)	448 (82)
S. glucose, mmol/l	**7.1 (4.8)**	**7.0 (4.0)**	6.5 (5.3)	6.4 (4.2)	8.0 (4.8)	7.9 (4.0)	**7.3 (2.7)**	**7.0 (2.8)**
HbA1c, percent	6.4 (1.4)	6.5 (1.6)	6.4 (1.4)	6.4 (1.5)	6.7 (1.6)	6.8 (1.6)	**6.0 (1.2)**	**6.1 (1.3)**
S. creatinine, µmol/l	**888 (283)**	**753 (229)**	**817 (241)**	**710 (206)**	**859 (307)**	**732 (245)**	**1057 (252)**	**881 (199)**
**Hemodialysis session**								
Pre-dialysis SBP, mm Hg	146 (23)	146 (24)	141 (22)	140 (24)	**149 (23)**	**151 (24)**	**152 (21)**	**149 (23)**
Treatment time, min	**239 (33)**	**228 (34)**	**246 (34)**	**235 (32)**	**225 (31)**	**211 (31)**	**244 (29)**	**240 (30)**
Blood flow rate, ml/min	**321 (94)**	**314 (93)**	**322 (58)**	**307 (56)**	**413 (63)**	**397 (65)**	**204 (32)**	**190 (32)**
IDWG, percent body weight	3.5 (1.8)	3.5 (1.9)	3.1 (1.7)	3.1 (1.9)	**3.6 (1.9)**	**3.5 (1.9)**	**4.1 (1.7)**	**4.3 (1.8)**
*Kt/V*	**1.4 (0.3)**	**1.6 (0.3)**	**1.4 (0.3)**	**1.6 (0.3)**	**1.4 (0.3)**	**1.6 (0.3)**	**1.3 (0.2)**	**1.5 (0.3)**
*Kt/V*<1.2, percent	**25.0**	**11.9**	**23.4**	**12.9**	**18.3**	**9.5**	**35.9**	**13.8**
Vascular access: AV fistula	**73.4**	**58.2**	**79.4**	**67.4**	**47.4**	**28.1**	**95.0**	**90.3**
Vascular access: AV graft	**14.4**	**23.4**	**6.8**	**12.2**	**33.9**	**46.1**	**4.2**	**8.8**
Vascular access: catheter	**12.2**	**18.4**	**13.8**	**20.4**	**18.7**	**25.9**	**0.8**	**0.9**
**Medication prescription**								
ESA	**85.4**	**91.1**	**86.8**	**91.4**	**89.5**	**93.5**	**77.3**	**86.7**
Phosphate binder	85.6	85.1	84.2	84.5	87.8	87.5	**85.3**	**82.2**
Vitamin D	52.4	51.9	45.5	45.3	56.7	55.6	60.2	59.9
Cinacalcet	**9.3**	**11.1**	**10.1**	**11.6**	**12.3**	**15.0**	4.5	4.8
Antihypertensives	**75.8**	**72.5**	**72.1**	**67.2**	80.5	78.6	**77.0**	**73.7**
Antibiotics	**4.7**	**5.8**	**4.8**	**5.7**	**7.2**	**8.6**	1.6	1.6
**Cause of ESRD**								
Diabetes	**27.2**	**28.8**	**19.7**	**20.9**	**37.1**	**43.6**	**29.2**	**21.3**
Glomerulonephritis, vasculitis	**28.0**	**26.1**	**24.9**	**21.3**	**14.2**	**14.4**	**50.6**	**55.2**
Hypertension	**17.8**	**15.3**	**16.9**	**12.2**	**30.5**	**26.6**	**4.0**	**3.6**
Other	**27.0**	**29.7**	**38.5**	**45.6**	**18.2**	**15.4**	**16.2**	**19.9**
**Comorbidities, percent**								
Diabetes	35.5	37.5	**28.6**	**30.2**	**47.8**	**54.8**	**32.9**	**23.9**
Coronary artery disease	**46.1**	**41.7**	**46.6**	**37.7**	**57.1**	**55.3**	**31.2**	**27.0**
Cerebrovascular disease	**17.4**	**16.7**	**18.2**	**16.4**	18.3	19.7	**14.8**	**12.4**
CHF	**30.6**	**31.4**	**30.0**	**27.8**	42.7	45.0	16.4	16.3
Hypertension	**80.3**	**78.5**	**80.0**	**77.0**	88.6	88.6	**70.3**	**64.7**
PVD	**29.6**	**23.7**	**34.2**	**24.8**	**32.7**	**28.9**	**16.6**	**12.6**
Other CVD	**36.1**	**35.6**	**39.6**	**38.3**	35.0	35.1	30.8	30.4
Cancer	**12.5**	**10.6**	**14.5**	**12.1**	**12.5**	**11.1**	**8.4**	**6.5**
GI bleed	**5.9**	**5.5**	5.3	4.8	8.0	7.4	4.6	3.9
Lung disease	**12.7**	**9.7**	**15.2**	**9.4**	**16.8**	**14.6**	2.7	2.2
Neurologic disorder	**10.5**	**11.8**	10.8	11.3	13.5	14.3	**6.0**	**8.6**
Psychologic disorder	**16.2**	**18.6**	**17.3**	**20.4**	24.4	24.8	3.6	4.4
Depression	**12.3**	**16.3**	**13.1**	**17.9**	**19.1**	**21.8**	**2.1**	**3.6**
Recurrent cellulitis	**9.4**	**8.4**	**9.9**	**8.7**	12.6	11.5	**4.4**	**2.6**
**Surgical interventions**								
Prior parathyroidectomy	**5.8**	**8.8**	**6.6**	**12.1**	**4.0**	**5.1**	**6.5**	**7.8**
Prior transplant	7.7	7.0	11.2	11.0	**7.4**	**5.4**	1.5	1.1
Transplant (during study)	**7.0**	**5.6**	**10.4**	**8.8**	**7.0**	**4.4**	0.6	0.9
**Cause of death** b								
CV	**48.2**	**46.5**	**43.7**	**40.9**	53.6	51.4	49.3	48.4
Infection	**16.4**	**15.1**	**18.8**	**15.9**	13.2	13.6	17.5	18.5
Other and unknown	**35.3**	**38.5**	**37.6**	**43.2**	33.2	35.0	33.2	33.1

Among patients in the initial prevalent cross-section of each DOPPS phase with time on dialysis >90 d. Bold indicates *p*<0.05 for men compared with women adjusted for DOPPS phase, country, US black race, and age. *p*-Values for black race were not adjusted for US black race, and *p*-values for age were not adjusted for age.

a Restricted to patients in facilities that routinely measure C-reactive protein (at least quarterly for 75% of patients).

b Among deaths with a listed cause. “Unknown” was indicated for 8% of deaths. 16% did not have listed cause.

AV, arteriovenous; CHF, congestive heart failure; CV, cardiovascular; CVD, cardiovascular disease; ESA, erythropoiesis-stimulating agent; ESRD, end-stage renal disease; GI, gastrointestinal; IDWG, interdialytic weight gain; PTH, parathyroid hormone; PVD, peripheral vascular disease; S., serum; SBP, systolic blood pressure.

Among laboratory values, serum parathyroid hormone (lower in men), C-reactive protein (higher in men), and serum creatinine levels (higher in men) showed clinically relevant, sex-dependent differences. These trends were consistent across regions, with the exception that serum parathyroid hormone in North America was equivalent in men and women (Table 2). Among hemodialysis treatment characteristics, treatment times were longer and blood flow rates higher in men than in women. However, hemodialysis adequacy, measured by *Kt/V* (small molecule clearance [*K*] times dialysis session length [*t*] divided by body water volume [*V*]), was lower in men, largely because of their higher weight (greater *V*). Vascular access also showed sex-dependent differences, with catheter use less frequent in men (12.2%) than in women (18.4%). Erythropoiesis-stimulating agents were less frequently prescribed for men (85.4%) than for women (91.1%), and so were antibiotics, while antihypertensives were more frequently prescribed for men (75.8% versus 72.5%).

Comorbidities showed several sex-dependent differences of statistical significance. The highest magnitude of these differences was observed for coronary artery disease (more frequent in men, by 4.4 percentage points) and depression (less frequent in men, by 4.0 percentage points). Diabetes was more prevalent (in both sexes) in North America than in the other DOPPS regions, and was diagnosed less frequently among men than among women in both Europe and North America (Table 2). Men had prior parathyroidectomy less frequently than women, but were more likely to have received a prior kidney transplant and to receive a kidney transplant during the course of the study. Causes of death were not strikingly different between men and women and did not show large regional discrepancies. All variables listed in Table 2 are also shown by country (rather than region) in Table S2.

### Characteristics of the Hemodialysis Population, by Sex and Age Group

As shown in Table 3, the overall trends for patient characteristic distributions in men versus women were not consistent throughout age groups. This was especially obvious for socioeconomic factors (marital status, education, and employment), where sex-specific differences were greater in higher age categories. Sex-specific differences in comorbidities also became more evident with increasing age. For example, the higher prevalence of coronary artery disease in men and depression in women was much more prominent in higher age categories.

**Table 3:** Patient characteristics, by sex and age group.

Characteristic	Age Group									
	18–44 y		45–54 y		55–64 y		65–74 y		75+ y	
	Men (*n* = 2,242)	Women (*n* = 1,499)	Men (*n* = 2,797)	Women (*n* = 1,822)	Men (*n* = 3,902)	Women (*n* = 2,863)	Men (*n* = 4,466)	Women (*n* = 3,482)	Men (*n* = 3,673)	Women (*n* = 3,036)
**Demographics**										
Age, years	36.0 (6.4)	35.7 (6.6)	50.0 (2.8)	50.0 (2.8)	59.8 (2.8)	59.8 (2.8)	69.5 (2.9)	69.6 (2.8)	80.0 (4.1)	80.1 (4.2)
Time on hemodialysis, years	6.3 (6.3)	6.1 (6.0)	6.7 (7.0)	6.8 (7.0)	**5.9 (6.4)**	**6.5 (6.7)**	**4.7 (4.8)**	**5.1 (5.5)**	**3.9 (3.7)**	**4.2 (3.9)**
Body mass index, kg/m^2^	24.0 (5.4)	24.3 (6.7)	**24.7 (5.4)**	**25.2 (7.3)**	**24.6 (5.3)**	**25.6 (7.1)**	**24.5 (4.8)**	**25.4 (6.5)**	23.8 (4.1)	23.9 (5.2)
Body mass index ≥30 kg/m^2^	**11.4**	**16.8**	**15.3**	**22.3**	**14.4**	**23.4**	**11.8**	**21.2**	**7.1**	**12.3**
Black race, percent	17.4	19.3	15.7	15.0	**10.6**	**14.5**	**7.1**	**11.9**	**4.8**	**7.4**
Married, percent	**32.0**	**37.9**	56.1	57.4	**71.7**	**61.7**	**77.2**	**52.2**	**72.7**	**31.9**
Employed, percent	**42.2**	**24.0**	**39.5**	**17.2**	**25.2**	**8.3**	**8.8**	**2.2**	3.3	1.0
Smoker, percent	**34.0**	**20.9**	**32.3**	**15.9**	**25.5**	**9.6**	**15.8**	**6.4**	**8.0**	**3.2**
Education less than high school, percent	35.2	36.8	36.8	40.0	**44.2**	**51.5**	**52.1**	**62.6**	**52.6**	**66.1**
**Laboratory values**										
S. phosphorus, mmol/l	**2.0 (0.7)**	**1.9 (0.6)**	1.9 (0.6)	1.9 (0.6)	1.8 (0.6)	1.8 (0.5)	1.7 (0.5)	1.7 (0.5)	**1.6 (0.5)**	**1.6 (0.5)**
S. calcium, mmol/l	2.3 (0.3)	2.3 (0.3)	**2.3 (0.2)**	**2.3 (0.2)**	**2.3 (0.2)**	**2.3 (0.2)**	**2.3 (0.2)**	**2.3 (0.2)**	**2.3 (0.2)**	**2.3 (0.2)**
S. PTH, ng/l	363 (402)	371 (413)	318 (341)	323 (354)	**266 (290)**	**304 (329)**	**238 (256)**	**268 (311)**	**228 (236)**	**240 (249)**
S. potassium, mmol/l	**5.1 (0.8)**	**5.1 (0.8)**	5.1 (0.8)	5.1 (0.8)	5.1 (0.8)	5.1 (0.8)	**5.0 (0.8)**	**5.0 (0.8)**	5.0 (0.7)	5.0 (0.8)
S. sodium, mmol/l	**138.4 (3.4)**	**137.3 (3.1)**	**138.2 (3.6)**	**137.7 (3.5)**	138.3 (3.5)	138.2 (3.6)	**138.5 (3.5)**	**138.1 (3.7)**	**138.4 (3.5)**	**138.0 (3.8)**
S. albumin, g/l	**39.9 (4.7)**	**38.4 (4.6)**	**38.9 (4.7)**	**37.9 (4.5)**	**38.2 (4.7)**	**37.5 (4.5)**	**37.6 (4.8)**	**37.0 (4.4)**	**36.8 (4.5)**	**36.4 (4.8)**
Hemoglobin, g/l	**113 (16)**	**111 (16)**	**112 (16)**	**109 (15)**	**112 (15)**	**109 (16)**	**112 (16)**	**111 (15)**	**114 (15)**	**112 (14)**
C-reactive protein, nmol/l^a^	104 (221)	101 (217)	99.5 (193.4)	94.7 (186.6)	96.6 (194.6)	91.8 (196.3)	125 (228)	119 (224)	**138 (224)**	**108 (196)**
Uric acid, µmol/l	**430 (96)**	**415 (87)**	**426 (93)**	**411 (86)**	**418 (94)**	**408 (90)**	**410 (91)**	**391 (89)**	**379 (86)**	**368 (86)**
S. glucose, mmol/l	**6.6 (5.1)**	**6.0 (3.4)**	6.8 (3.9)	7.1 (4.3)	7.3 (4.6)	7.2 (4.0)	7.3 (5.4)	7.2 (4.0)	7.1 (4.5)	7.1 (4.1)
HbA1c, percent	6.7 (1.8)	6.5 (2.0)	6.8 (1.6)	7.0 (2.1)	6.5 (1.5)	6.6 (1.5)	**6.3 (1.3)**	**6.5 (1.3)**	6.1 (1.2)	6.1 (1.3)
S. creatinine, µmol/l	**1,084 (301)**	**905 (254)**	**1007 (284)**	**842 (229)**	**918 (267)**	**791 (216)**	**824 (240)**	**709 (197)**	**723 (203)**	**640 (188)**
**Hemodialysis session**										
Pre-dialysis SBP, mm Hg	**148 (22)**	**142 (23)**	148 (23)	148 (25)	**148 (23)**	**147 (24)**	**146 (23)**	**147 (24)**	**142 (23)**	**144 (24)**
Treatment time, min	**243 (34)**	**228 (36)**	**246 (34)**	**236 (35)**	**243 (33)**	**232 (32)**	**238 (32)**	**227 (32)**	**229 (32)**	**220 (33)**
Blood flow rate, ml/min	**340 (90)**	**328 (89)**	**325 (98)**	**309 (97)**	**312 (97)**	**308 (97)**	**313 (93)**	**314 (91)**	**326 (87)**	**315 (90)**
IDWG, percent body weight	4.0 (2.1)	4.0 (2.2)	3.9 (1.9)	3.8 (2.0)	3.7 (1.8)	3.6 (1.9)	**3.3 (1.7)**	**3.3 (1.9)**	3.0 (1.6)	3.1 (1.8)
*Kt/V*	**1.4 (0.3)**	**1.6 (0.3)**	**1.4 (0.3)**	**1.5 (0.3)**	**1.4 (0.3)**	**1.5 (0.3)**	**1.4 (0.3)**	**1.6 (0.3)**	**1.4 (0.3)**	**1.6 (0.3)**
*Kt/V*<1.2, percent	**25.5**	**10.3**	**28.5**	**13.3**	**25.0**	**11.8**	**25.7**	**11.2**	**21.2**	**12.7**
Vascular access: AV fistula	**75.2**	**60.4**	**75.2**	**61.6**	**75.8**	**61.1**	**73.9**	**57.0**	**67.9**	**54.0**
Vascular access: AV graft	**14.3**	**23.1**	**14.7**	**22.7**	**14.1**	**23.5**	**13.9**	**24.9**	**15.2**	**22.2**
Vascular access: catheter	**10.6**	**16.5**	**10.2**	**15.7**	**10.1**	**15.5**	**12.2**	**18.1**	**16.9**	**23.8**
**Medication prescription**										
ESA	**85.9**	**90.7**	**80.2**	**89.3**	**83.0**	**90.6**	**86.4**	**91.1**	**90.3**	**92.9**
Phosphate binder	**91.9**	**89.5**	89.5	88.9	87.5	87.9	84.6	84.3	77.7	79.0
Vitamin D	54.2	54.3	**57.0**	**52.7**	53.1	53.1	50.5	51.6	49.4	49.4
Cinacalcet	14.2	16.2	14.9	13.8	**10.0**	**12.6**	**7.3**	**9.8**	**5.8**	**8.2**
Antihypertensives	**75.8**	**66.7**	**75.8**	**69.1**	**77.8**	**73.3**	**77.0**	**74.3**	72.1	74.6
Antibiotics	**5.3**	**8.3**	**5.2**	**6.6**	**4.6**	**5.9**	4.5	5.0	4.4	5.2
**Cause of ESRD**										
Diabetes	**14.8**	**15.0**	**25.1**	**25.4**	**34.9**	**32.6**	**32.8**	**35.6**	**21.5**	**26.1**
Glomerulonephritis, vasculitis	**37.1**	**42.3**	**35.6**	**36.8**	**29.4**	**26.9**	**24.6**	**19.7**	**19.2**	**18.5**
Hypertension	**12.9**	**10.2**	**14.2**	**9.4**	**13.3**	**11.4**	**17.6**	**15.1**	**28.5**	**25.4**
Other	**35.2**	**32.4**	**25.2**	**28.3**	**22.5**	**29.1**	**25.0**	**29.6**	**30.8**	**30.0**
**Comorbidities, percent**										
Diabetes	18.5	18.7	30.5	31.8	**43.5**	**39.2**	42.4	46.1	**32.5**	**38.3**
Coronary artery disease	23.0	23.2	**35.4**	**31.6**	**47.8**	**41.5**	**52.9**	**46.8**	**58.3**	**51.1**
Cerebrovascular disease	4.8	5.9	11.1	10.4	**16.8**	**15.2**	22.0	20.8	**24.9**	**22.8**
CHF	18.6	19.0	24.7	24.1	**30.4**	**29.6**	**34.4**	**34.7**	38.0	39.9
Hypertension	**78.7**	**73.5**	**78.5**	**74.0**	**79.8**	**77.8**	**81.8**	**80.3**	81.3	82.2
PVD	9.4	9.5	**20.4**	**17.5**	**30.7**	**23.4**	**37.0**	**28.8**	**38.6**	**28.9**
Other CVD	17.2	17.5	24.9	23.9	33.6	32.3	41.6	40.5	**52.2**	**49.0**
Cancer	2.6	3.6	**5.9**	**7.8**	10.3	10.8	**15.0**	**12.0**	**22.7**	**13.9**
GI bleed	3.8	3.2	5.9	4.5	5.5	5.2	6.5	6.3	6.9	6.6
Lung disease	3.4	4.7	7.7	6.7	**11.0**	**9.6**	**15.9**	**10.8**	**20.1**	**13.0**
Neurologic disorder	10.8	12.3	8.2	8.3	7.3	8.7	9.7	10.7	16.1	17.8
Psychologic disorder	22.2	21.9	18.5	20.4	**16.0**	**20.2**	**14.6**	**17.8**	**12.7**	**15.4**
Depression	14.8	17.7	**12.3**	**16.8**	**11.5**	**17.7**	**12.3**	**15.9**	**11.5**	**14.4**
Recurrent cellulitis	6.3	5.2	8.7	8.9	**10.4**	**9.1**	**11.1**	**9.6**	8.7	7.7
**Surgical interventions**										
Prior parathyroidectomy	11.0	13.2	**9.2**	**11.8**	**7.0**	**10.1**	**3.3**	**8.0**	**1.8**	**4.2**
Prior transplant	23.3	22.1	13.2	13.1	6.8	6.8	3.2	3.1	0.7	0.4
Transplant (during study)	18.4	17.4	11.8	10.8	**7.8**	**6.4**	3.2	2.0	0.2	0.1
**Cause of death** b										
CV	40.5	46.8	46.8	40.5	**56.5**	**49.9**	47.0	45.0	45.0	45.1
Infection	15.3	18.9	18.9	15.3	**15.2**	**14.0**	17.0	15.2	17.0	15.0
Other and unknown	44.3	34.2	34.2	44.3	**28.4**	**36.1**	36.0	39.9	38.0	40.0

Among patients in the initial prevalent cross-section of each DOPPS phase with time on dialysis >90 d. Bold indicates *p*<0.05 for men compared with women adjusted for DOPPS phase, country, US black race, and age. *p*-Values for black race did not include Japan and were adjusted for region instead of country, and *p*-values for age were not adjusted for age.

a Restricted to patients in facilities that routinely measure C-reactive protein (at least quarterly for 75% of patients).

b Among deaths with a listed cause. “Unknown” was indicated for 8% of deaths. 16% did not have listed cause.

AV, arteriovenous; CHF, congestive heart failure; CV, cardiovascular; CVD, cardiovascular disease; ESA, erythropoiesis-stimulating agent; ESRD, end-stage renal disease; GI, gastrointestinal; IDWG, interdialytic weight gain; PTH, parathyroid hormone; PVD, peripheral vascular disease; S., serum; SBP, systolic blood pressure.

### Adjusted Male-to-Female Mortality of the Hemodialysis Population

The unadjusted adult male-to-female mortality rate ratios shown in Figure 2 might have been influenced by sex-dependent differences in the characteristics of the study population. When we used Cox models to determine the adult male-to-female mortality risk in the DOPPS, we found that the hazard ratio (HR) and 95% confidence interval of mortality of men (versus women) moved from 1.03 (95% CI 0.99–1.08, unadjusted baseline model, stratified by country and DOPPS phase) to 1.09 (95% CI 1.04–1.14) overall after adjusting for age and time on dialysis. Regional differences are shown in Figure 3. This result indicates that men have a slightly, but significantly, higher mortality risk than women at similar age and time on dialysis.

Subsequent adjustments for treatment-related factors and case mix altered the male-to-female mortality risk only marginally, with the exception of the adjustment for serum creatinine, which strongly increased the male-to-female mortality risk. Since the sequence of adding adjustments may modify the results, we conducted a sensitivity analysis where we adjusted first for case mix before adjusting for treatment-related factors. The findings were consistent: adding adjustments for treatment time, hemodialysis catheter use, hemoglobin, diabetes, serum albumin, and serum creatinine showed a shift to the right (increasing the HR for men versus women), while adding adjustments for cardiovascular comorbidities, other comorbidities, and body mass index showed a shift to the left (decreasing the HR; Figure S1).

### Interaction Analyses

Men's and women's mortality HRs (associated with hemodialysis characteristics showing at least a close to significant sex interaction) are presented in Figure 4. Of these characteristics, hemodialysis catheter use displayed the largest difference in mortality risk between men (HR = 1.11, in comparison to no catheter) and women (HR = 1.33), interaction *p*<0.001. The higher mortality risk associated with coronary artery disease, cardiovascular disease, diabetes, and neurologic disorder was also lower for men than for women. Similarly, the lower mortality risk associated with body mass index was even lower for men than for women. However, the mortality advantage associated with phosphate binder use and vitamin D use was significant, and greater for women, while the 95% confidence interval crossed 1.0 for men. These trends were largely consistent across regions (Table S3).

## Discussion

The principal findings of this large, international analysis of sex-dependent differences in the characteristics, treatment, and outcomes of patients with end-stage renal disease were as follows: (1) fewer women than men were undergoing hemodialysis treatment, consistent with national hemodialysis registry data, despite higher proportions of women in the general population across all age groups; (2) the survival advantage that women have over men in the general population was markedly diminished in hemodialysis patients; (3) despite substantial cross-sectional differences between men and women on hemodialysis, adjustments altered the crude male-to-female mortality rate ratio only slightly; and (4) certain hemodialysis characteristics showed a significant sex interaction with mortality and with further study may become targets to improve outcomes. As further elaborated below, the findings of our paper are important for researchers and caretakers in our field to explore and potentially minimize barriers for women to receive hemodialysis treatment, and to improve dialysis practices where outcomes differ by sex.

The finding that fewer women than men were undergoing hemodialysis treatment in all DOPPS countries could in principle be related to differences in treatment modality for end-stage renal disease (in-center hemodialysis versus home hemodialysis versus peritoneal dialysis). However, the United States Renal Data System [23] showed, like the Canadian registry [20], that both the incidence and prevalence of peritoneal dialysis and home hemodialysis were higher in men than women, and that more men had preemptively received a kidney transplant.

Differences in sex-dependent survival on hemodialysis could also have contributed to the greater proportion of men among prevalent hemodialysis patients observed in the present analysis. Women's survival on dialysis, however, was equal to or better than men's survival. Although amount of time on hemodialysis did not show a significant sex interaction, sex-dependent differences in early hemodialysis mortality might nevertheless exert an effect, as we excluded patients who had dialyzed <90 d at study entry for the derivation of this study's prevalent cross-section. Data in Table 1 confirm that the percentage of women (compared to men) on hemodialysis is markedly lower both at dialysis initiation and for the cross-section (i.e., in incident and prevalent patients). If only the healthiest women received hemodialysis, they would be expected to survive longer, and the percentages for women in the prevalent cross-section would be expected to show an increase from incident patients. Table 1 demonstrates such a trend, and may thus allow the aforementioned speculation.

Interestingly, chronic kidney disease among individuals without end-stage renal disease has been found to be more frequent among women in the National Health and Nutrition Examination Survey (NHANES) [23] and in the majority of population-based studies [11], at least when eGFR is used to estimate impaired renal function [26]. Specifically, NHANES data showed for 2005–2010 that the percentage of adult patients with eGFR <60 ml/min/1.73 m^2^ was substantially higher in women (7.7%) than in men (5.6%). Regarding the transition to renal failure, the United States Renal Data System annual data report showed for Medicare patients that advanced chronic kidney disease (stage 4 and 5) without dialysis was also more common in women than in men [23]. Acute renal failure accounts for a very small fraction of reported end-stage renal disease causes (1.2% in 2011 according to the United States Renal Data System 2013 report [23]). Thus, this cause would play a negligible role. The progression of renal disease has been shown to be faster among men [27],[28], and risk factors such as age, body mass index, and plasma glucose have been shown to contribute to men's progression to end-stage renal disease to a greater extent than for comparable women [29], which might partly explain why the proportion of hemodialysis patients that are women increases with age (Figure 1).

However, the present study identified large national differences in the proportion of dialysis patients that are women, even within the same age groups, e.g., ranging for older hemodialysis patients (≥75 y) from as low as 31.9% women in Australia-New Zealand to 48.9% in Canada (Table 1). These findings were confirmed by registry data and suggest that hemodialysis initiation may largely be influenced by psychosocioeconomic issues [30]. As shown in Tables 2 and 3, sex differences included a larger proportion of women with a lower education level, which has been associated with diabetes [31] as well as cardiovascular events [32]. Women have also been shown to be less aware of chronic kidney disease [12] and, moreover, to initiate hemodialysis later than men [33]. These findings are consistent with previously shown sex-specific differences at hemodialysis initiation [34],[35]. Moreover, and in accordance with the findings of the present study, recent analysis of US data at Arbor Research Collaborative for Health showed that the adjusted eGFR was 1.07 ml/min/1.73 m^2^ lower in women than in men at dialysis initiation (95% CI 1.03–1.10 ml/min/1.73 m^2^) [36].

For women, later dialysis initiation and higher death rates before hemodialysis initiation may be a consequence of not seeking timely medical care, and possible differential decision making regarding dialysis initiation. Iseki et al. performed mass screening with dipstick urinalysis and blood pressure measurements and found, consistent with our study, that fewer women than men received renal replacement therapy during 10 y of follow-up in their Japanese cohort [37]. Such an approach may, however, not capture the true need for hemodialysis. In the United States, a large analysis among adult members of an integrated health care system provider suggested near equal access (and possibly similar need) for hemodialysis for both sexes [38]. Both of these analyses, however, excluded patients with no outpatient measurements of serum creatinine. The competing impacts of sex-specific kidney disease prevalence, progression, awareness, and subsequent hemodialysis initiation should therefore be studied prospectively in patients with chronic kidney disease while tracking the transition to hemodialysis. The recently initiated international Chronic Kidney Disease Outcomes and Practice Patterns Study (CKDopps) is designed to shed light on access to end-stage renal disease care and mortality in advanced chronic kidney disease.

Three previous studies [13],[39],[40] have analyzed the mortality risk of adult male versus female hemodialysis patients. Carrero et al., using the large dataset of the European Renal Association–European Dialysis and Transplant Association registry, reported that younger women have a higher risk for dying of non-cardiovascular causes [13]. This finding could not be confirmed in the present study of prevalent hemodialysis patients, where causes of death among those aged 18–44 y did not differ significantly between men and women (Table 3; note that we were unable to split the data into more granular age categories). The essential finding of both previous studies, however, that the mortality advantage of women in the general population is essentially cancelled out in hemodialysis patients, was confirmed in the present analysis (Figures 2 and 3), which is by its large international sample size adequately powered to control for many measured confounders.

In an attempt to explain this finding [39], analyses from the general population may be helpful in demonstrating that the risk associated with diabetes and cardiovascular disease is greater among women than among men [41]–[43]. Our interaction analyses in the DOPPS (Figure 4) agreed with these studies from the general population, and indicated that the mortality risk associated with diabetes, coronary artery disease, and cardiovascular disease was higher among adult female than male hemodialysis patients. Moreover, higher body mass index, which is well known to be associated with better survival in hemodialysis patients [44],[45], was slightly “less protective” among women than men in our analysis. However, given a hemodialysis mortality rate 10 to 20 times higher than in the general population [46]–[48], hemodialysis may be viewed as a “great equalizer.” Differences between men and women that have an important impact in the general population might therefore lose importance in patients with end-stage renal disease.

We examined whether sex-dependent differences in patient and hemodialysis characteristics might exert a small or large effect on mortality by determining HRs for the adult male-to-female mortality risk in hemodialysis patients with stepwise-increasing levels of adjustment (Figure 3). There was no question that baseline adjustments (for age and time on dialysis) were necessary, to deal with the most basic confounders. These adjustments moved the HR of mortality of men (versus women) from 1.03 (in the unadjusted baseline model, stratified by country and DOPPS phase) to a statistically significant HR of 1.09. Further adjustments, however, were a matter for debate. Adjustments for modifiable variables were useful in determining the potential value of changing hemodialysis practices to improve sex-dependent patient care. Adjustment for serum creatinine, mostly an indicator of muscle mass in end-stage renal disease [44], should be considered largely non-modifiable because of the known average differences in muscle mass by sex, and is therefore primarily of theoretical interest. Case-mix adjustments and adjustments for all variables for which men and women are biologically different (most of them non-modifiable) might thus be misleading by creating a “unisex association with mortality.”

Interestingly, adjustments for case mix and modifiable factors except for serum creatinine altered the adult male-to-female mortality risk only slightly (Figure 3), and the order of adjustment did not substantially influence the results (Figure S1), indicating that women were not strikingly healthier than men. A relatively greater impact was observed for *Kt/V*, hemodialysis catheter use, diabetes, cardiovascular comorbidities, and body mass index. Hemodialysis catheter use, diabetes, cardiovascular comorbidities, and body mass index also showed a significant sex association with mortality, and may be worth further study. The difference in adult male-to-female mortality exerted by adjustment for hemodialysis dose (i.e., *Kt/V*) is likely a consequence of *Kt/V* being genuinely higher in women (Tables 2 and 3). The sex-dependent mortality associated with hemodialysis dose, however, varies when *Kt/V* is scaled to body surface area, as recently addressed more thoroughly in a DOPPS analysis [49].

The small excess mortality risk for men in Japan and some European countries was not detected in North America and some other European countries. Future studies may clarify the extent to which the sex-specific differences in mortality risk are related to the general population's background mortality differences [50] versus related to better care for women in the respective hemodialysis facilities or other factors. Catheter use for hemodialysis vascular access could be reduced for women [51], particularly outside Japan (Tables 2 and 3). Traditionally, a fear of smaller vessels in women may have prevented some nephrologists from considering arteriovenous fistulae in female hemodialysis patients [52],[53]. Published data using duplex ultrasonography, however, demonstrated that vessel diameter does not differ between the sexes [54], as emphasized by recent research [55]. Surgical training is likely the key to both arteriovenous fistula placement and survival, as recent observational studies did not observe sex differences in arteriovenous fistula failure, perhaps reflecting an improvement in both technique and physician experience [56],[57]. Such a development would be encouraging and points to an opportunity for improving vascular access for women [51]. The results by sex in our intention-to-treat analyses among patients on dialysis >90 d differ from those of the CHOICE study, which used as-treated analyses for incident patients and showed that catheter use was associated with mortality risk among men but not among women [58]. Thus, hemodialysis vascular access by sex deserves more study to also consider whether our sex-specific findings on vascular access and mortality are partly explained by selection.

The present study on hemodialysis patients is shedding light on several sex-dependent issues that have also been addressed in the general population [59]–[61]. Among these issues, smoking and marriage prevalence differed by sex in hemodialysis patients, and may have an effect on outcomes. Our finding of higher rates of clinician-diagnosed depression in women agrees with a previous DOPPS analysis showing that women have a significantly higher prevalence of depressive symptoms according to the Center for Epidemiologic Studies Depression Scale [62]. Access to transplantation has also been previously shown to be lower in women [63], as reinforced by the data presented in Tables 2 and 3.

Several limitations need to be acknowledged. The presented analyses of adjusted mortality risk can show only associations, not causation, and can thus merely hint at the mechanisms that render mortality rates similar in men and women on hemodialysis. Likewise, our descriptive findings of hemodialysis prevalence by sex cannot answer *why* the prevalence of hemodialysis treatment is higher for men than women. However, the large national differences we identified strongly suggest that the reasons go beyond biological ones. After careful review of the present data and the literature, we believe the data suggest that women with end-stage renal disease are less likely than men to receive hemodialysis treatment, perhaps because of psychosocioeconomic factors. It also is possible that women are less likely than men to receive hemodialysis because the severity of their disease is not recognized by their caregivers, they are less aware of their disease and the degree of its severity [12], or they are more reluctant to undergo treatment. The present large study followed a suggestion made many years ago that hemodialysis mortality for women should be analyzed internationally [64]. Despite limitations, it may now open a window of subsequent research opportunities and possibilities to improve patient care.

In conclusion, we showed among patients treated with hemodialysis for end-stage renal disease that women differ from men in a vast number of variables, some of which appear related to biology, some to patient care or to society. The finding that the general survival advantage for women is virtually lost for all adult age groups of individuals on dialysis is striking. Variation among the DOPPS regions in the very small survival advantage for women on hemodialysis might be partly explained by similar variations in the general population. The impact of different levels of adjustments on adult male-to-female mortality as well as other sex-related factors (in our statistical interaction studies) points to higher catheter-related mortality risk for women than observed for men, and suggests an opportunity to improve hemodialysis practices. Whether men and women differ by dialysis initiation and chronic kidney disease care is perhaps the most important question raised by the present study. This question is not novel, as national data have been available for decades, but may not previously have been asked as clearly as by the present analysis with a large sample size and international perspective. Future international studies should concentrate on considering sex differences as a factor for treating patients with end-stage renal disease, not only for improving outcomes, but also for equalizing women's access to renal replacement therapy.

## Supporting Information

Figure S1 **Adjusted hazard ratios for the adult male-to-female mortality risk in hemodialysis patients, by region (order of case mix and “modifiable” adjustments reversed from** **Figure 3** **).** ^a^Stratified by country (including US black race and US non-black race) and phase; *n* = 36,216 patients (*n* = 8,258 deaths) among patients with time on dialysis >90 d dialyzing 3× weekly. ^b^Coronary artery disease, cerebrovascular disease, congestive heart failure, hypertension, peripheral vascular disease, other cardiovascular disease. ^c^Cancer, gastrointestinal bleed, lung disease, neurologic disorder, psychologic disorder, recurrent cellulitis. ^d^European countries = Belgium, France, Germany, Italy, Spain, Sweden, UK. ^e^Education, employment, marital status, smoking status, predialysis systolic blood pressure, blood flow rate, serum potassium, medication prescriptions (erythropoiesis-stimulating agent, phosphate binder, vitamin D, antihypertensive, antibiotic), prior parathyroidectomy, and prior transplant. A/NZ, Australia/New Zealand; BMI, body mass index; CV, cardiovascular; HD, hemodialysis; IDWG, interdialytic weight gain; N. America, North America; PTH, parathyroid hormone.
(TIF)

Table S1 **Percentage of patients that are women in the hemodialysis population from national registry data compared to DOPPS.**
(DOCX)

Table S2 **Patient characteristics, by sex and country.**
(DOCX)

Table S3 **Analysis of sex interaction in the associations between hemodialysis patient characteristics and mortality, by region.**
(DOCX)

Checklist S1 **STROBE Statement checklist of items that should be included in reports of observational studies.** Responses to the STROBE Statement recommendations are provided in bold italic.
(DOCX)

## Abbreviations

DOPPS: Dialysis Outcomes and Practice Patterns Study
eGFR: estimated glomerular filtration rate
HR: hazard ratio
MDRD: Modification of Diet in Renal Disease Study
NHANES: National Health and Nutrition Examination Survey
