# Supplementary figures and images for: Sex-Specific Differences in Hemodialysis Prevalence and Practices and the Male-to-Female Mortality Rate: The Dialysis Outcomes and Practice Patterns Study (DOPPS)
Source: PLoS Med. 2014 Oct 28;11(10):e1001750. doi: 10.1371/journal.pmed.1001750 (PMC4211675; doi:10.1371/journal.pmed.1001750)

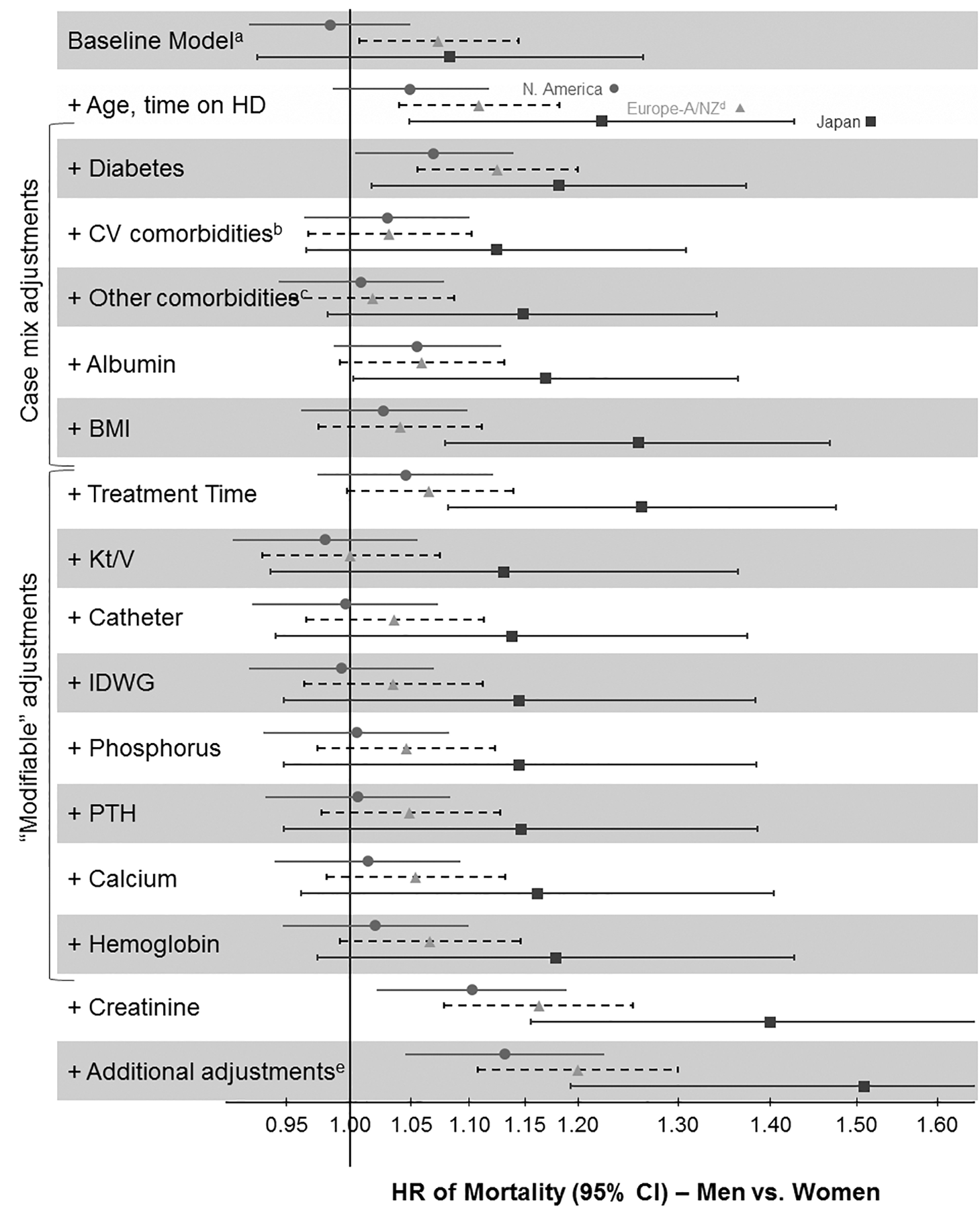

Supplement: Figure S1 — Adjusted hazard ratios for the adult male-to-female mortality risk in hemodialysis patients, by region (order of case mix and “modifiable” adjustments reversed from Figure 3 ). aStratified by country (including US black race and US non-black race) and phase; n = 36,216 patients (n = 8,258 deaths) among patients with time on dialysis >90 d dialyzing 3× weekly. bCoronary artery disease, cerebrovascular disease, congestive heart failure, hypertension, peripheral vascular disease, other cardiovascular disease. cCancer, gastrointestinal bleed, lung disease, neurologic disorder, psychologic disorder, recurrent cellulitis. dEuropean countries = Belgium, France, Germany, Italy, Spain, Sweden, UK. eEducation, employment, marital status, smoking status, predialysis systolic blood pressure, blood flow rate, serum potassium, medication prescriptions (erythropoiesis-stimulating agent, phosphate binder, vitamin D, antihypertensive, antibiotic), prior parathyroidectomy, and prior transplant. A/NZ, Australia/New Zealand; BMI, body mass index; CV, cardiovascular; HD, hemodialysis; IDWG, interdialytic weight gain; N. America, North America; PTH, parathyroid hormone. (TIF) [file pmed.1001750.s001.tif]
