# Supplementary material for: Sex-Specific Differences in Hemodialysis Prevalence and Practices and the Male-to-Female Mortality Rate: The Dialysis Outcomes and Practice Patterns Study (DOPPS)
Source: PLoS Med. 2014 Oct 28;11(10):e1001750. doi: 10.1371/journal.pmed.1001750 (PMC4211675; doi:10.1371/journal.pmed.1001750)
Supplement: Table S1 — Percentage of patients that are women in the hemodialysis population from national registry data compared to DOPPS. (DOCX) [file pmed.1001750.s002.docx]

**Supporting Information**

**Table S1: Percentage of women in the hemodialysis population from national registry data compared to DOPPS**

|  | **% Women** | |
| --- | --- | --- |
| **Country** | **Registry** | **DOPPS^g^** |
| Australia-New Zealand^a^ | 41 | 41 |
| Belgium^b^ | 40 | 42 |
| Canada^c^ | 42 | 44 |
| France^b^ | 40 | 41 |
| Germany^d^ | -- | 40 |
| Italy^d^ | -- | 41 |
| Japan^e^ | 38 | 37 |
| Spain^b^ | 38 | 39 |
| Sweden^b^ | 36 | 38 |
| United Kingdom^b^ | 39 | 41 |
| United States^f^ | 44 | 45 |

1. Australia and New Zealand Dialysis and Transplant Registry 2012 Annual report
2. ERA-EDTA Registry 2011 Annual Report
3. Canadian Organ Replacement Register 2012 Annual Report
4. Public registry not available
5. Japanese Society for Dialysis Therapy “Overview of Regular Dialysis Treatment in Japan (As of 31 December 2010)”
6. U.S. Renal Data System 2012 Annual Report
7. DOPPS 4 (2009) initial prevalent cross-section of patients (n=28,926)
