# Supplementary material for: Sex-Specific Differences in Hemodialysis Prevalence and Practices and the Male-to-Female Mortality Rate: The Dialysis Outcomes and Practice Patterns Study (DOPPS)
Source: PLoS Med. 2014 Oct 28;11(10):e1001750. doi: 10.1371/journal.pmed.1001750 (PMC4211675; doi:10.1371/journal.pmed.1001750)
Supplement: Table S2 — Patient characteristics, by sex and country. (DOCX) [file pmed.1001750.s003.docx]

**Table S2: Patient characteristics, by sex and country**

|  | **Europe-Australia/New Zealand** | | | | | | | | | | | | | | | | | | | | | | | | | |  | **North America** | | | | |  | **Japan** | |
| --- | --- | --- | --- | --- | --- | --- | --- | --- | --- | --- | --- | --- | --- | --- | --- | --- | --- | --- | --- | --- | --- | --- | --- | --- | --- | --- | --- | --- | --- | --- | --- | --- | --- | --- | --- |
|  | **Australia** | |  | **Belgium** | |  | **France** | |  | **Germany** | |  | **Italy** | |  | **New Zealand** | |  | **Spain** | |  | **Sweden** | |  | **United Kingdom** | |  | **Canada** | |  | **United States** | |  |  | |
| **Characteristic** | **Male** | **Female** |  | **Male** | **Female** |  | **Male** | **Female** |  | **Male** | **Female** |  | **Male** | **Female** |  | **Male** | **Female** |  | **Male** | **Female** |  | **Male** | **Female** |  | **Male** | **Female** |  | **Male** | **Female** |  | **Male** | **Female** |  | **Male** | **Female** |
|  | (n=734) | (n=482) |  | (n=780) | (n=594) |  | (n=983) | (n=787) |  | (n=1146) | (n=863) |  | (n=1105) | (n=815) |  | (n=72) | (n=71) |  | (n=1217) | (n=881) |  | (n=804) | (n=453) |  | (n=995) | (n=701) |  | (n=738) | (n=596) |  | (n=4452) | (n=3857) |  | (n=4083) | (n=2633) |
| **Demographics** |  |  |  |  |  |  |  |  |  |  |  |  |  |  |  |  |  |  |  |  |  |  |  |  |  |  |  |  |  |  |  |  |  |  |  |
| Age, years | 63.5(14.5) | 61.6(13.9) |  | 67.7(13.7) | 68.6(13.1) |  | **62.9(15.5)** | **65.1(14.9)** |  | 62.2(13.8) | 63.2(15.0) |  | 64.7(13.3) | 65.1(13.8) |  | 57.7(14.3) | 54.7(13.5) |  | **62.9(15.5)** | **64.5(15.0)** |  | 65.2(14.2) | 64.7(14.4) |  | 60.0(16.7) | 59.7(16.2) |  | 62.6(15.2) | 64.0(15.4) |  | **60.3(15.5)** | **62.3(15.0)** |  | **60.8(12.5)** | **62.2(12.7)** |
| Time on dialysis, years | 5.0(5.1) | 5.3(5.3) |  | 3.9(4.7) | 4.2(4.9) |  | **6.2(7.0)** | **6.9(7.5)** |  | 4.5(4.9) | 4.7(4.9) |  | **5.6(5.8)** | **6.6(6.7)** |  | 4.9(4.0) | 5.3(4.8) |  | 4.8(5.3) | 5.2(5.5) |  | 4.5(5.2) | 4.7(5.5) |  | 4.8(5.2) | 5.1(5.4) |  | 4.6(5.0) | 4.7(5.0) |  | 3.7(3.9) | 3.8(3.9) |  | **7.8(6.9)** | **8.7(6.9)** |
| Body mass index, kg/m^2^ | 26.3(5.6) | 26.5(6.7) |  | **24.8(4.1)** | **26.1(5.8)** |  | **24.0(4.3)** | **24.7(6.1)** |  | 25.7(4.3) | 25.6(5.7) |  | 24.3(4.1) | 24.3(5.3) |  | 27.4(5.3) | 30.4(8.5) |  | 24.4(3.9) | 24.7(4.9) |  | 25.1(4.5) | 25.3(5.8) |  | 25.2(4.8) | 25.5(6.3) |  | 26.3(5.7) | 27.0(6.8) |  | **26.0(5.8)** | **27.5(7.3)** |  | **21.2(2.9)** | **20.1(3.2)** |
| Body mass index ≥ 30 kg/m^2^ | 22.5 | 25.2 |  | **11.1** | **21.6** |  | **8.4** | **18.4** |  | **13.7** | **19.6** |  | **7.7** | **13.7** |  | **28.6** | **44.8** |  | **8.4** | **12.7** |  | **13.1** | **18.8** |  | **14.4** | **20.2** |  | **20.9** | **28.6** |  | **20.4** | **31.7** |  | 0.9 | 0.8 |
| Black race, % | 0.5 | 0.2 |  | 3.5 | 2.5 |  | 3.6 | 2.8 |  | 0.5 | 0.2 |  | 0.7 | 0.2 |  | 0.0 | 1.4 |  | 0.7 | 0.3 |  | 0.5 | 2.0 |  | 5.6 | 7.1 |  | 5.4 | 5.2 |  | **34.8** | **38.6** |  | - | - |
| Married | **65.7** | **49.5** |  | **69.4** | **49.4** |  | **68.2** | **47.7** |  | **69.3** | **48.4** |  | **70.8** | **51.1** |  | **67.1** | **44.3** |  | **68.5** | **52.8** |  | **55.9** | **45.5** |  | **61.3** | **50.3** |  | **59.0** | **40.1** |  | **55.3** | **36.9** |  | **76.1** | **65.3** |
| Employed | **12.3** | **9.1** |  | **7.9** | **2.1** |  | **14.2** | **6.7** |  | **13.0** | **7.3** |  | **15.7** | **4.9** |  | 26.5 | 17.9 |  | **12.6** | **4.3** |  | **15.1** | **11.3** |  | **17.4** | **11.0** |  | **12.4** | **5.4** |  | **12.4** | **7.0** |  | **45.2** | **12.6** |
| Smoker | 11.9 | 10.0 |  | **20.1** | **7.5** |  | **19.9** | **9.2** |  | **19.8** | **8.9** |  | **17.4** | **6.6** |  | **14.8** | **11.1** |  | **14.3** | **5.9** |  | 13.4 | 11.1 |  | **19.5** | **14.7** |  | 21.4 | 19.1 |  | **20.6** | **11.0** |  | **32.1** | **6.3** |
| Ed. less than high school | **51.0** | **62.7** |  | **44.4** | **57.9** |  | **63.8** | **76.9** |  | **75.7** | **84.4** |  | **62.6** | **78.0** |  | 41.4 | 46.5 |  | **61.2** | **79.0** |  | **62.5** | **69.3** |  | **48.6** | **55.8** |  | 47.2 | 51.3 |  | **33.7** | **38.9** |  | **29.3** | **37.6** |
| **Laboratory Values** |  |  |  |  |  |  |  |  |  |  |  |  |  |  |  |  |  |  |  |  |  |  |  |  |  |  |  |  |  |  |  |  |  |  |  |
| S. Phosphorus, mg/dL | 5.3(1.8) | 5.3(1.8) |  | 5.0(1.7) | 5.1(1.5) |  | **5.0(1.8)** | **5.2(1.6)** |  | 6.1(2.1) | 6.1(2.0) |  | **4.9(1.6)** | **5.1(1.6)** |  | 5.6(2.0) | 5.5(1.7) |  | 5.2(1.6) | 5.1(1.6) |  | **5.2(1.8)** | **5.5(1.7)** |  | 5.3(1.9) | 5.3(1.8) |  | 5.2(1.7) | 5.2(1.6) |  | 5.8(1.9) | 5.7(1.8) |  | **5.7(1.6)** | **5.6(1.5)** |
| S. Calcium, mg/dL | 9.3(0.8) | 9.4(0.9) |  | **9.0(0.8)** | **9.2(0.9)** |  | **9.1(1.0)** | **9.2(1.0)** |  | **9.0(0.9)** | **9.1(1.0)** |  | 9.4(1.4) | 9.4(1.3) |  | 9.6(0.9) | 9.6(0.8) |  | 9.4(0.9) | 9.5(0.8) |  | **9.4(0.8)** | **9.5(1.0)** |  | 9.5(0.8) | 9.5(0.9) |  | 9.1(0.8) | 9.1(0.8) |  | **9.2(0.9)** | **9.2(0.9)** |  | **9.1(0.9)** | **9.3(0.9)** |
| S. PTH, pg/mL | **350(355)** | **405(456)** |  | 246(242) | 250(248) |  | **257(271)** | **309(325)** |  | 248(265) | 263(280) |  | **263(258)** | **311(337)** |  | 454(381) | 581(494) |  | 275(295) | 300(339) |  | 264(259) | 254(252) |  | 319(385) | 325(359) |  | 341(359) | 334(374) |  | 318(346) | 314(343) |  | 189(212) | 198(219) |
| S. Potassium, mEq/L | 5.1(0.7) | 5.0(0.7) |  | 5.0(0.8) | 4.9(0.7) |  | 5.0(0.8) | 5.0(0.8) |  | 5.3(0.8) | 5.4(0.8) |  | 5.5(0.8) | 5.5(0.9) |  | 4.8(0.8) | 5.0(0.8) |  | 5.4(0.8) | 5.5(0.8) |  | 4.8(0.7) | 4.8(0.8) |  | 5.0(0.8) | 5.1(0.8) |  | 4.8(0.7) | 4.8(0.7) |  | 4.8(0.7) | 4.9(0.7) |  | 5.1(0.8) | 5.0(0.8) |
| S. Sodium, mEq/L | 137.9(3.5) | 137.0(3.6) |  | **137.8(3.7)** | **137.4(4.2)** |  | **137.7(3.3)** | **137.2(3.5)** |  | **138.5(3.8)** | **138.1(4.1)** |  | 138.5(3.7) | 138.3(3.7) |  | **137.3(3.8)** | **136.2(2.8)** |  | **138.3(3.7)** | **137.9(3.7)** |  | **138.7(3.4)** | **137.9(3.3)** |  | **138.1(3.3)** | **137.4(3.3)** |  | **138.0(3.5)** | **137.7(3.4)** |  | **138.2(3.6)** | **137.9(3.6)** |  | 139.1(3.2) | 139.0(3.2) |
| S. Albumin, g/dL | **3.7(0.5)** | **3.7(0.4)** |  | 3.8(0.6) | 3.8(0.5) |  | **3.8(0.5)** | **3.7(0.4)** |  | **4.0(0.6)** | **4.0(0.6)** |  | **3.9(0.6)** | **3.8(0.6)** |  | 3.9(0.5) | 3.8(0.4) |  | **3.9(0.5)** | **3.8(0.5)** |  | 3.5(0.4) | 3.5(0.5) |  | **3.8(0.5)** | **3.7(0.5)** |  | **3.6(0.4)** | **3.5(0.5)** |  | **3.8(0.4)** | **3.7(0.4)** |  | **3.8(0.4)** | **3.8(0.4)** |
| Hemoglobin, g/dL | **11.9(1.5)** | **11.6(1.4)** |  | 11.6(1.3) | 11.7(1.3) |  | **11.4(1.6)** | **11.2(1.5)** |  | 11.5(1.4) | 11.4(1.4) |  | **11.4(1.5)** | **11.1(1.6)** |  | 11.4(1.8) | 11.3(1.8) |  | **11.7(1.6)** | **11.5(1.6)** |  | **12.0(1.4)** | **11.8(1.3)** |  | **11.4(1.6)** | **11.1(1.6)** |  | **11.6(1.3)** | **11.5(1.4)** |  | **11.5(1.5)** | **11.3(1.4)** |  | **10.3(1.3)** | **9.9(1.3)** |
| C-reactive protein, mg/L^a^ | **21.7(30.4)** | **15.8(23.5)** |  | 14.9(23.9) | 13.2(23.7) |  | **15.6(26.4)** | **12.2(19.1)** |  | 13.7(22.2) | 14.7(25.6) |  | 14.2(24.5) | 12.2(20.6) |  | - | - |  | 14.1(24.7) | 12.9(24.3) |  | 17.7(25.6) | 17.3(25.5) |  | 18.0(24.0) | 16.8(27.2) |  | - | - |  | - | - |  | 5.3(14.6) | 4.4(13.6) |
| Uric acid, mg/dL | 6.2(1.3) | 6.0(1.5) |  | **6.9(1.4)** | **6.6(1.4)** |  | 6.5(1.4) | 6.3(1.4) |  | **7.0(1.6)** | **6.6(1.5)** |  | **6.4(1.4)** | **6.2(1.3)** |  | - | - |  | **6.4(1.5)** | **6.0(1.3)** |  | 5.7(1.4) | 5.5(1.2) |  | 6.1(1.6) | 6.0(1.3) |  | **6.2(1.5)** | **5.8(1.3)** |  | **6.7(1.5)** | **6.3(1.4)** |  | 7.6(1.4) | 7.5(1.4) |
| S. Glucose, mg/dL | 137(109) | 130(85) |  | 105(121) | 102(62) |  | 102(61) | 117(112) |  | 122(61) | 116(46) |  | 115(98) | 112(82) |  | 116(42) | 141(91) |  | 111(56) | 112(53) |  | 145(144) | 130(61) |  | 126(124) | 126(100) |  | **144(99)** | **131(71)** |  | 144(83) | 146(72) |  | **131(48)** | **125(50)** |
| HbA1c, % | 6.5(1.5) | 6.7(1.7) |  | 6.3(1.1) | 6.3(1.2) |  | 6.8(1.5) | 6.9(1.4) |  | 6.4(1.2) | 6.4(1.3) |  | 6.9(1.3) | 6.9(1.5) |  | 6.6(2.1) | 7.0(1.8) |  | 6.4(1.6) | 6.4(1.6) |  | 5.7(1.4) | 5.7(1.6) |  | 6.7(1.4) | 6.7(1.7) |  | 6.9(1.9) | 7.1(1.8) |  | 6.7(1.5) | 6.7(1.6) |  | **6.0(1.2)** | **6.1(1.3)** |
| Creatinine, mg/dL | **8.9(2.5)** | **7.9(2.2)** |  | **9.1(3.1)** | **7.9(2.6)** |  | **9.4(2.6)** | **8.1(2.2)** |  | **9.3(3.0)** | **8.2(2.6)** |  | **10.0(2.5)** | **8.6(2.2)** |  | 9.3(2.9) | 8.3(2.2) |  | **9.3(2.7)** | **8.0(2.2)** |  | **8.2(2.4)** | **7.2(2.0)** |  | **9.3(2.8)** | **8.0(2.5)** |  | **8.8(2.8)** | **7.6(2.3)** |  | **9.9(3.6)** | **8.4(2.8)** |  | **12.0(2.8)** | **10.0(2.3)** |
| **Dialysis Session** |  |  |  |  |  |  |  |  |  |  |  |  |  |  |  |  |  |  |  |  |  |  |  |  |  |  |  |  |  |  |  |  |  |  |  |
| Pre-dialysis SBP, mmHg | 145(22) | 146(23) |  | 141(21) | 143(24) |  | 141(22) | 140(23) |  | 137(20) | 136(21) |  | **137(22)** | **135(23)** |  | 148(22) | 153(26) |  | 138(21) | 136(23) |  | 144(25) | 142(25) |  | 146(24) | 147(25) |  | 146(22) | 146(24) |  | **150(23)** | **152(24)** |  | **152(21)** | **149(23)** |
| Treatment time, min | **270(32)** | **257(32)** |  | **236(22)** | **232(24)** |  | **248(33)** | **241(30)** |  | **262(37)** | **251(35)** |  | **229(25)** | **223(26)** |  | 264(28) | 253(23) |  | **229(24)** | **220(26)** |  | **266(35)** | **246(31)** |  | **237(32)** | **223(31)** |  | **236(24)** | **225(28)** |  | **224(32)** | **209(31)** |  | **244(29)** | **240(30)** |
| Blood flow rate, mL/min | **319(42)** | **306(43)** |  | **328(78)** | **316(79)** |  | **315(48)** | **306(49)** |  | **286(51)** | **273(46)** |  | **316(50)** | **301(45)** |  | 311(37) | 298(44) |  | **344(47)** | **332(48)** |  | **337(60)** | **314(55)** |  | **334(61)** | **312(59)** |  | **379(55)** | **362(62)** |  | **419(63)** | **402(64)** |  | **204(32)** | **190(32)** |
| IDWG, % body weight | 2.8(1.5) | 2.9(1.6) |  | **2.7(1.6)** | **2.4(1.7)** |  | 3.6(1.8) | 3.6(1.8) |  | 2.7(1.8) | 2.6(2.0) |  | 3.9(1.7) | 4.1(1.9) |  | 2.5(1.2) | 2.5(1.5) |  | 3.2(1.6) | 3.2(1.7) |  | 2.7(1.7) | 2.7(1.8) |  | 2.6(1.6) | 2.6(1.8) |  | 3.2(1.7) | 3.2(1.7) |  | **3.7(2.0)** | **3.5(1.9)** |  | **4.1(1.7)** | **4.3(1.8)** |
| Kt/V | **1.5(0.3)** | **1.7(0.3)** |  | **1.4(0.3)** | **1.5(0.3)** |  | **1.4(0.3)** | **1.6(0.3)** |  | **1.3(0.3)** | **1.5(0.3)** |  | **1.3(0.3)** | **1.5(0.3)** |  | **1.3(0.2)** | **1.4(0.3)** |  | **1.4(0.3)** | **1.6(0.3)** |  | **1.5(0.3)** | **1.6(0.3)** |  | **1.4(0.3)** | **1.5(0.3)** |  | **1.5(0.3)** | **1.6(0.3)** |  | **1.4(0.3)** | **1.6(0.3)** |  | **1.3(0.2)** | **1.5(0.3)** |
| Kt/V < 1.2, % | **15.5** | **5.8** |  | **26.3** | **17.8** |  | **20.8** | **8.4** |  | **31.8** | **15.5** |  | **30.1** | **19.2** |  | 25.8 | 16.4 |  | **22.7** | **12.3** |  | **13.4** | **8.2** |  | **24.2** | **12.7** |  | **16.6** | **8.4** |  | **18.6** | **9.7** |  | **35.9** | **13.8** |
| Vascular Access |  |  |  |  |  |  |  |  |  |  |  |  |  |  |  |  |  |  |  |  |  |  |  |  |  |  |  |  |  |  |  |  |  |  |  |
| AV Fistula | **83.2** | **70.7** |  | **66.4** | **51.0** |  | **82.6** | **70.7** |  | **86.2** | **76.0** |  | **87.6** | **78.5** |  | 73.9 | 62.0 |  | **81.2** | **67.7** |  | **66.3** | **48.1** |  | **75.5** | **64.4** |  | **61.1** | **40.5** |  | **45.2** | **26.2** |  | **95.0** | **90.3** |
| AV Graft | **8.5** | **20.4** |  | **2.0** | **3.8** |  | **8.8** | **15.9** |  | **7.9** | **14.8** |  | **3.4** | **5.9** |  | 7.2 | 16.9 |  | **8.0** | **11.5** |  | **10.5** | **19.4** |  | **5.0** | **9.3** |  | **10.0** | **13.3** |  | **37.8** | **51.1** |  | **4.2** | **8.8** |
| Catheter | **8.2** | **8.9** |  | **31.6** | **45.3** |  | **8.5** | **13.4** |  | **6.0** | **9.2** |  | **9.0** | **15.6** |  | 18.8 | 21.1 |  | **10.8** | **20.8** |  | **23.2** | **32.6** |  | **19.5** | **26.3** |  | **29.0** | **46.1** |  | **17.0** | **22.7** |  | **0.8** | **0.9** |
| **Medication prescription** |  |  |  |  |  |  |  |  |  |  |  |  |  |  |  |  |  |  |  |  |  |  |  |  |  |  |  |  |  |  |  |  |  |  |  |
| ESA | **86.9** | **92.1** |  | **95.1** | **97.5** |  | **79.6** | **86.0** |  | **83.3** | **87.1** |  | **82.8** | **90.4** |  | 71.8 | 80.3 |  | **88.9** | **94.7** |  | **92.5** | **96.1** |  | 90.2 | 91.9 |  | **91.1** | **95.7** |  | **89.3** | **93.1** |  | **77.3** | **86.7** |
| Phosphate binder | 91.7 | 91.5 |  | 86.5 | 87.3 |  | 78.7 | 80.4 |  | 82.7 | 81.4 |  | 81.6 | 83.1 |  | 76.1 | 83.1 |  | 88.7 | 86.9 |  | **87.8** | **91.6** |  | 79.6 | 80.1 |  | 94.2 | 91.6 |  | 86.8 | 86.9 |  | **85.3** | **82.2** |
| Vitamin D | 52.3 | 53.7 |  | 33.3 | 34.0 |  | 27.9 | 28.3 |  | 53.2 | 52.9 |  | 46.1 | 48.7 |  | 72.2 | 66.2 |  | 43.1 | 42.6 |  | 53.4 | 54.3 |  | 52.8 | 50.8 |  | 48.8 | 43.9 |  | 58.0 | 57.4 |  | 60.2 | 59.9 |
| Cinacalcet | 9.4 | 7.3 |  | 5.0 | 7.0 |  | **11.1** | **15.1** |  | 12.3 | 12.3 |  | 10.1 | 13.8 |  | 0.0 | 0.0 |  | **13.2** | **19.4** |  | 11.5 | 10.2 |  | 7.4 | 5.2 |  | 3.0 | 4.7 |  | **15.0** | **17.9** |  | 4.5 | 4.8 |
| Antihypertensives | 70.8 | 67.2 |  | 72.2 | 69.4 |  | **72.5** | **69.4** |  | **87.4** | **83.1** |  | **59.8** | **56.0** |  | **68.1** | **83.1** |  | **61.1** | **52.8** |  | **85.4** | **80.4** |  | **71.6** | **64.4** |  | **83.7** | **78.9** |  | 80.0 | 78.5 |  | **77.0** | **73.7** |
| Antibiotics | 5.2 | 6.1 |  | 7.5 | 9.1 |  | 4.8 | 4.7 |  | 5.1 | 5.3 |  | 2.5 | 2.8 |  | 2.8 | 2.8 |  | **1.3** | **2.6** |  | **9.7** | **13.9** |  | 5.1 | 6.5 |  | 8.8 | 10.4 |  | **6.9** | **8.4** |  | 1.6 | 1.6 |
| **Cause of ESRD** |  |  |  |  |  |  |  |  |  |  |  |  |  |  |  |  |  |  |  |  |  |  |  |  |  |  |  |  |  |  |  |  |  |  |  |
| Diabetes | 28.7 | 25.5 |  | **21.8** | **27.1** |  | **15.2** | **19.5** |  | **26.3** | **24.2** |  | **13.3** | **13.9** |  | 36.6 | 50.0 |  | **17.7** | **19.0** |  | **24.4** | **24.8** |  | 12.9 | 14.9 |  | 34.8 | 30.6 |  | **37.6** | **45.7** |  | **29.2** | **21.3** |
| Glomerulonephritis, Vasculitis | 29.9 | 26.2 |  | **17.8** | **12.6** |  | **28.0** | **24.5** |  | **28.1** | **19.7** |  | **26.0** | **24.4** |  | 18.3 | 28.6 |  | **21.7** | **19.2** |  | **23.6** | **21.4** |  | 23.8 | 21.6 |  | 20.6 | 21.2 |  | **13.1** | **13.3** |  | **50.6** | **55.2** |
| Hypertension | 11.9 | 8.5 |  | **25.1** | **17.5** |  | **18.3** | **13.7** |  | **16.0** | **12.5** |  | **16.9** | **11.5** |  | 9.9 | 2.9 |  | **16.3** | **10.7** |  | **19.7** | **13.7** |  | 13.1 | 10.8 |  | 20.5 | 22.6 |  | **32.2** | **27.3** |  | **4.0** | **3.6** |
| Other | 29.5 | 39.7 |  | **35.3** | **42.8** |  | **38.6** | **42.3** |  | **29.6** | **43.6** |  | **43.9** | **50.1** |  | 35.2 | 18.6 |  | **44.3** | **51.1** |  | **32.3** | **40.1** |  | 50.2 | 52.7 |  | 24.1 | 25.7 |  | **17.1** | **13.8** |  | **16.2** | **19.9** |
| **Comorbidities** |  |  |  |  |  |  |  |  |  |  |  |  |  |  |  |  |  |  |  |  |  |  |  |  |  |  |  |  |  |  |  |  |  |  |  |
| Diabetes | 38.4 | 40.5 |  | 33.6 | 38.2 |  | 23.3 | 27.8 |  | 34.9 | 33.4 |  | 19.8 | 21.7 |  | 46.5 | 52.9 |  | 25.6 | 27.6 |  | 37.1 | 36.9 |  | 20.9 | 21.1 |  | 46.5 | 43.8 |  | **48.1** | **56.5** |  | **32.9** | **23.9** |
| Coronary artery disease | **64.2** | **51.5** |  | **54.7** | **44.8** |  | **39.2** | **30.9** |  | **56.4** | **49.4** |  | **35.7** | **30.7** |  | 52.8 | 36.6 |  | **34.9** | **28.1** |  | **52.2** | **41.5** |  | **44.6** | **33.7** |  | 56.2 | 52.8 |  | **57.2** | **55.7** |  | **31.2** | **27.0** |
| Cerebrovascular disease | **21.5** | **15.1** |  | 24.6 | 25.6 |  | **17.0** | **12.4** |  | **19.1** | **16.0** |  | 16.4 | 16.1 |  | 13.9 | 11.3 |  | 17.3 | 15.1 |  | 19.9 | 19.4 |  | 12.5 | 15.0 |  | 20.5 | 21.5 |  | 18.0 | 19.5 |  | **14.8** | **12.4** |
| CHF | 42.0 | 37.3 |  | **39.8** | **34.0** |  | **32.5** | **28.3** |  | **29.0** | **25.8** |  | 15.0 | 16.6 |  | 15.3 | 26.8 |  | 33.3 | 36.9 |  | **32.5** | **26.9** |  | **23.7** | **19.9** |  | 37.8 | 42.6 |  | 43.5 | 45.4 |  | 16.4 | 16.3 |
| Hypertension | 88.1 | 83.4 |  | 81.5 | 82.2 |  | 80.9 | 77.5 |  | **85.3** | **82.5** |  | **70.8** | **64.5** |  | **65.3** | **77.1** |  | **82.8** | **79.4** |  | 82.6 | 77.8 |  | 72.0 | 71.8 |  | 89.4 | 89.0 |  | 88.4 | 88.5 |  | **70.3** | **64.7** |
| PVD | **42.9** | **31.1** |  | **43.8** | **31.6** |  | **40.0** | **30.5** |  | **37.2** | **25.3** |  | **29.5** | **20.4** |  | **19.4** | **33.8** |  | **34.5** | **24.3** |  | **29.9** | **22.1** |  | **20.8** | **13.9** |  | **36.2** | **29.2** |  | **32.2** | **28.9** |  | **16.6** | **12.6** |
| Other CVD | 38.4 | 33.8 |  | 50.3 | 51.9 |  | **43.8** | **40.8** |  | **40.0** | **37.5** |  | 34.9 | 38.5 |  | 38.9 | 32.4 |  | 39.6 | 42.8 |  | 43.3 | 37.7 |  | **30.2** | **23.3** |  | 43.5 | 46.5 |  | 33.6 | 33.3 |  | 30.8 | 30.4 |
| Cancer | 13.8 | 13.9 |  | 16.9 | 17.3 |  | **17.8** | **13.2** |  | 14.6 | 12.5 |  | **11.6** | **8.6** |  | 11.1 | 7.1 |  | **13.1** | **10.4** |  | 17.2 | 14.3 |  | 12.6 | 10.4 |  | 17.7 | 16.1 |  | **11.7** | **10.3** |  | **8.4** | **6.5** |
| GI Bleed | 6.6 | 4.8 |  | 5.5 | 5.3 |  | 3.8 | 3.7 |  | 5.2 | 4.7 |  | 4.4 | 4.8 |  | 4.2 | 5.6 |  | 5.5 | 5.2 |  | 5.4 | 5.5 |  | 6.2 | 4.7 |  | 6.3 | 5.1 |  | 8.3 | 7.8 |  | 4.6 | 3.9 |
| Lung disease | 16.6 | 15.1 |  | **24.2** | **13.4** |  | **15.3** | **6.9** |  | **12.7** | **9.3** |  | **14.2** | **9.8** |  | 9.9 | 11.3 |  | **22.1** | **7.8** |  | 8.3 | 8.2 |  | 8.5 | 7.0 |  | 18.3 | 19.3 |  | **16.5** | **13.9** |  | 2.7 | 2.2 |
| Neurologic disorder | 13.3 | 9.6 |  | 14.5 | 16.4 |  | 9.4 | 10.1 |  | 13.3 | 12.3 |  | 9.1 | 11.2 |  | 11.1 | 19.7 |  | 9.9 | 10.6 |  | **10.8** | **13.9** |  | 7.0 | 7.1 |  | 12.5 | 12.8 |  | 13.7 | 14.6 |  | **6.0** | **8.6** |
| Psychologic disorder | 24.2 | 21.3 |  | **18.7** | **24.1** |  | **16.3** | **18.9** |  | **14.2** | **19.5** |  | **16.9** | **20.2** |  | 5.6 | 4.2 |  | **17.7** | **24.6** |  | **16.2** | **21.6** |  | 17.2 | 15.6 |  | 19.1 | 18.8 |  | 25.3 | 25.7 |  | 3.6 | 4.4 |
| Depression | 19.1 | 18.7 |  | **15.5** | **20.9** |  | **9.4** | **15.5** |  | **9.1** | **15.8** |  | **13.4** | **18.4** |  | 2.8 | 2.8 |  | **13.6** | **22.9** |  | **12.9** | **19.0** |  | 14.8 | 13.8 |  | 14.5 | 17.1 |  | **19.9** | **22.6** |  | **2.1** | **3.6** |
| Recurrent cellulitus | **16.1** | **10.0** |  | 13.7 | 13.8 |  | 7.1 | 7.2 |  | 13.3 | 11.9 |  | 6.5 | 6.0 |  | 15.3 | 15.7 |  | 6.6 | 7.1 |  | 11.5 | 8.9 |  | 7.2 | 5.4 |  | 13.1 | 10.9 |  | 12.5 | 11.5 |  | **4.4** | **2.6** |
| **Surgical Interventions** |  |  |  |  |  |  |  |  |  |  |  |  |  |  |  |  |  |  |  |  |  |  |  |  |  |  |  |  |  |  |  |  |  |  |  |
| Prior parathyroidectomy | **11.3** | **20.7** |  | **4.6** | **8.8** |  | **10.0** | **16.7** |  | **6.3** | **11.1** |  | **3.2** | **7.0** |  | 16.9 | 15.5 |  | **4.6** | **7.5** |  | **6.6** | **13.5** |  | **7.1** | **15.9** |  | **7.7** | **10.1** |  | **3.4** | **4.3** |  | **6.5** | **7.8** |
| Prior transplant | 10.0 | 11.0 |  | 6.1 | 4.6 |  | 14.1 | 13.0 |  | 7.3 | 7.5 |  | 5.7 | 5.7 |  | **12.5** | **5.6** |  | **15.5** | **17.5** |  | 13.2 | 12.2 |  | 17.1 | 16.3 |  | 11.7 | 8.9 |  | **6.7** | **4.8** |  | 1.5 | 1.1 |
| Transplant (during study) | 7.6 | 9.3 |  | 10.6 | 8.9 |  | 9.4 | 7.2 |  | 8.6 | 7.5 |  | **7.0** | **4.4** |  | 6.9 | 2.8 |  | 15.9 | 12.6 |  | 12.3 | 14.3 |  | 11.5 | 9.1 |  | 8.3 | 6.5 |  | **6.7** | **4.0** |  | 0.6 | 0.9 |
| **Cause of Death^b^** |  |  |  |  |  |  |  |  |  |  |  |  |  |  |  |  |  |  |  |  |  |  |  |  |  |  |  |  |  |  |  |  |  |  |  |
| CV | 49.7 | 43.4 |  | 40.7 | 34.5 |  | 47.1 | 40.4 |  | 46.0 | 43.4 |  | 44.3 | 43.2 |  | 33.3 | 47.4 |  | 38.0 | 45.6 |  | 48.4 | 43.0 |  | 37.8 | 30.6 |  | 36.1 | 34.7 |  | 55.8 | 53.6 |  | 49.3 | 48.4 |
| Infection | 15.6 | 14.5 |  | 18.7 | 20.8 |  | 17.6 | 13.5 |  | 20.0 | 19.4 |  | 10.5 | 9.3 |  | 40.0 | 15.8 |  | 22.9 | 14.5 |  | 21.5 | 18.2 |  | 20.0 | 16.7 |  | 12.3 | 11.1 |  | 13.3 | 13.9 |  | 17.5 | 18.5 |
| Other and unknown | 34.7 | 42.1 |  | 40.7 | 44.6 |  | 35.3 | 46.2 |  | 34.0 | 37.1 |  | 45.2 | 47.5 |  | 26.7 | 36.8 |  | 39.1 | 39.9 |  | 30.0 | 38.8 |  | 42.2 | 52.8 |  | 51.6 | 54.2 |  | 30.8 | 32.5 |  | 33.2 | 33.1 |

Among patients in the initial prevalent cross-section of each DOPPS phase with time on dialysis > 90 days

a. Restricted to patients in facilities that routinely measure CRP (at least quarterly for 75% of patients)

b. Among deaths with a listed cause. ‘Unknown’ was indicated for 8% of deaths. 16% did not have listed cause.

**Bold** indicates p < 0.05 for men compared with women adjusted for DOPPS phase, country, US black race, and age. P-values for black race were not adjusted for US black race, and p-values for age were not adjusted for age.

Ed.=Education, S.=serum, PTH=Parathyroid Hormone, SBP=Systolic Blood Pressure, IDWG=Interdialytic Weight Gain, Kt/V=hemodialysis dose, AV=arteriovenous, ESA=Erythropoiesis-Stimulating Agent, CHF=Congestive Heart Failure, PVD=Peripheral Vascular Disease, CVD=Cardiovascular Disease, GI=Gastrointestinal, CV=Cardiovascular, US=United States.
