# Supplementary material for: Sex-Specific Differences in Hemodialysis Prevalence and Practices and the Male-to-Female Mortality Rate: The Dialysis Outcomes and Practice Patterns Study (DOPPS)
Source: PLoS Med. 2014 Oct 28;11(10):e1001750. doi: 10.1371/journal.pmed.1001750 (PMC4211675; doi:10.1371/journal.pmed.1001750)
Supplement: Table S3 — Analysis of sex interaction in the associations between hemodialysis patient characteristics and mortality, by region. (DOCX) [file pmed.1001750.s004.docx]

**Table S3: Analysis of sex interaction in the associations between hemodialysis patient characteristics and mortality, by region**

|  |  | **Overall** | |  | **Japan** | |  | **Europe-A/NZ** | |  | **North America** | |
| --- | --- | --- | --- | --- | --- | --- | --- | --- | --- | --- | --- | --- |
| **Variable** | **Sex** | **HR (95% CI)** | **p-value** |  | **HR (95% CI)** | **p-value** |  | **HR (95% CI)** | **p-value** |  | **HR (95% CI)** | **p-value** |
| Catheter use | W | 1.33(1.22-1.45) | <.001 |  | 1.95(0.95-3.97) | 0.260 |  | 1.38(1.23-1.55) | 0.143 |  | 1.28(1.13-1.45) | 0.01 |
|  | M | 1.11(1.02-1.21) |  |  | 0.99(0.44-2.21) |  |  | 1.24(1.11-1.40) |  |  | 1.03(0.91-1.17) |  |
| Time on HD, per year | W | 1.01(1.00-1.02) | 0.011 |  | 1.02(1.00-1.03) | 0.174 |  | 1.01(1.00-1.02) | 0.052 |  | 1.02(1.01-1.03) | 0.599 |
|  | M | 1.02(1.01-1.02) |  |  | 1.03(1.02-1.04) |  |  | 1.02(1.01-1.03) |  |  | 1.02(1.01-1.03) |  |
| BMI, per 5 kg/m^2^ | W | 0.88(0.85-0.91) | 0.067 |  | 0.64(0.53-0.77) | 0.562 |  | 0.87(0.82-0.92) | 0.457 |  | 0.89(0.86-0.93) | 0.313 |
|  | M | 0.85(0.82-0.88) |  |  | 0.69(0.56-0.86) |  |  | 0.84(0.79-0.89) |  |  | 0.87(0.82-0.91) |  |
| Single pool Kt/V, per 0.5 | W | 0.82(0.77-0.88) | 0.113 |  | 0.60(0.47-0.76) | 0.001 |  | 0.83(0.76-0.92) | 0.274 |  | 0.85(0.78-0.94) | 0.717 |
|  | M | 0.89(0.84-0.95) |  |  | 0.93(0.73-1.20) |  |  |  |  |  | 0.88(0.80-0.98) |  |
| Diabetes | W | 1.23(1.14-1.32) | 0.133 |  | 1.46(1.10-1.93) | 0.988 |  | 1.28(1.15-1.43) | 0.149 |  | 1.14(1.02-1.27) | 0.589 |
|  | M | 1.16(1.08-1.24) |  |  | 1.47(1.18-1.84) |  |  | 1.16(1.04-1.28) |  |  | 1.11(1.01-1.22) |  |
| Coronary artery disease | W | 1.21(1.13-1.30) | 0.017 |  | 1.39(1.08-1.78) | 0.606 |  | 1.24(1.12-1.38) | 0.040 |  | 1.16(1.05-1.28) | 0.286 |
|  | M | 1.10(1.03-1.17) |  |  | 1.30(1.08-1.58) |  |  | 1.08(0.99-1.18) |  |  | 1.10(0.99-1.22) |  |
| Other CVD | W | 1.29(1.20-1.39) | 0.013 |  | 1.39(1.09-1.76) | 0.275 |  | 1.28(1.15-1.43) | 0.516 |  | 1.26(1.14-1.40) | 0.006 |
|  | M | 1.15(1.08-1.23) |  |  | 1.18(0.95-1.47) |  |  | 1.22(1.12-1.33) |  |  | 1.05(0.95-1.16) |  |
| Neurologic disorder | W | 1.43(1.30-1.56) | 0.068 |  | 1.66(1.25-2.20) | 0.181 |  | 1.39(1.21-1.60) | 0.577 |  | 1.34(1.17-1.53) | 0.175 |
|  | M | 1.29(1.18-1.40) |  |  | 1.36(1.04-1.78) |  |  | 1.34(1.19-1.51) |  |  | 1.18(1.04-1.35) |  |
| Prior transplant | W | 0.58(0.48-0.71) | 0.148 |  | 3.38(1.05-10.89) | 0.202 |  | 0.55(0.42-0.72) | 0.116 |  | 0.54(0.39-0.74) | 0.335 |
|  | M | 0.69(0.59-0.80) |  |  | 1.33(0.48-3.67) |  |  | 0.68(0.56-0.83) |  |  | 0.64(0.51-0.82) |  |
| Phosphate binder use | W | 0.82(0.75-0.91) | 0.053 |  | 0.87(0.64-1.19) | 0.641 |  | 0.80(0.70-0.92) | 0.037 |  | 0.86(0.75-0.99) | 0.572 |
|  | M | 0.93(0.86-1.02) |  |  | 0.93(0.71-1.22) |  |  | 0.98(0.88-1.09) |  |  | 0.92(0.78-1.07) |  |
| Vitamin D use | W | 0.91(0.84-0.99) | 0.103 |  | 0.97(0.71-1.31) | 0.912 |  | 0.87(0.76-1.00) | 0.227 |  | 0.94(0.84-1.05) | 0.226 |
|  | M | 0.98(0.90-1.06) |  |  | 0.97(0.79-1.19) |  |  | 0.96(0.86-1.08) |  |  | 1.01(0.91-1.12) |  |
| Age, per 10 years | W | 1.37(1.33-1.41) | 0.361 |  | 1.68(1.49-1.90) | 0.041 |  | 1.41(1.35-1.48) | 0.879 |  | 1.31(1.26-1.36) | 0.098 |
|  | M | 1.34(1.30-1.38) |  |  | 1.43(1.31-1.56) |  |  | 1.36(1.30-1.42) |  |  | 1.32(1.26-1.37) |  |
| GI bleed | W | 1.21(1.07-1.37) | 0.159 |  | 1.83(1.11-3.02) | 0.038 |  | 1.09(0.89-1.34) | 0.595 |  | 1.27(1.09-1.49) | 0.402 |
|  | M | 1.09(0.97-1.22) |  |  | 0.92(0.60-1.40) |  |  | 1.03(0.87-1.22) |  |  | 1.17(1.00-1.38) |  |
| Creatinine, per mg/dL | W | 0.92(0.90-0.93) | 0.215 |  | 0.81(0.76-0.86) | <.001 |  | 0.93(0.91-0.96) | 0.213 |  | 0.93(0.91-0.95) | 0.341 |
|  | M | 0.92(0.91-0.94) |  |  | 0.91(0.88-0.95) |  |  | 0.91(0.90-0.93) |  |  | 0.93(0.92-0.95) |  |
| SBP, per mmHg | W | 0.95(0.92-0.98) | 0.912 |  | 0.96(0.86-1.07) | 0.233 |  | 0.97(0.93-1.02) | 0.016 |  | 0.92(0.88-0.96) | 0.147 |
|  | M | 0.94(0.92-0.97) |  |  | 1.05(0.96-1.15) |  |  | 0.90(0.87-0.94) |  |  | 0.96(0.92-1.00) |  |

P-value is for interaction with sex, shown for variables with p<0.15 overall or in any region (n=37 interactions in each region and overall tested). Adjusted for all variables listed in Tables 2 and 3 in addition to variables listed in Figure 2.

Abbreviations: HR=Hazard ratio, CI=Confidence Interval, BMI=Body Mass Index, CVD=Cardiovascular Disease, GI=Gastrointestinal, CV=Cardiovascular, SBP=predialysis systolic blood pressure
