# Supplementary material for: Sex-Specific Differences in Hemodialysis Prevalence and Practices and the Male-to-Female Mortality Rate: The Dialysis Outcomes and Practice Patterns Study (DOPPS)
Source: PLoS Med. 2014 Oct 28;11(10):e1001750. doi: 10.1371/journal.pmed.1001750 (PMC4211675; doi:10.1371/journal.pmed.1001750)
Supplement: Checklist S1 — STROBE Statement checklist of items that should be included in reports of observational studies. Responses to the STROBE Statement recommendations are provided in bold italic. (DOCX) [file pmed.1001750.s005.docx]

STROBE Statement—checklist of items that should be included in reports of observational studies

|  | Item No | Recommendation |
| --- | --- | --- |
| **Title and abstract** | 1 | (*a*) Indicate the study’s design with a commonly used term in the title or the abstract |
|  |  | (*b*) Provide in the abstract an informative and balanced summary of what was done and what was found  ***Response:***  ***a) The Dialysis Outcomes and Practice Patterns Study (DOPPS) is an international prospective cohort study, as mentioned in the Methods section of our manuscript. Its design has previously been published (as referenced in the manuscript). The study title has been included in the title of the manuscript as well as in the Abstract.***  ***b)An informative and balanced summary of what was done and what was found is provided in the abstract of the present manuscript.*** |
| Introduction | | |
| Background/rationale | 2 | Explain the scientific background and rationale for the investigation being reported  ***Response:***  ***In our Introduction, we describe the large discrepancy between an encouragement to publish study data by sex, and the paucity of available literature.*** |
| Objectives | 3 | State specific objectives, including any prespecified hypotheses  ***Response:***  ***We clearly state our prespecified objectives, namely to identify patient variables or hemodialysis practices that can be modified in order to improve the care of women and men with end-stage renal disease by assessing (1) hemodialysis prevalence among study participants, overall and by country, (2) national differences in sex-dependent hemodialysis patient mortality, (3) sex-dependent differences in hemodialysis characteristics, and (4) the presence of a sex interaction in the associations between hemodialysis characteristics and mortality at the end of our Introduction.*** |
| Methods | | |
| Study design | 4 | Present key elements of study design early in the paper  ***Response:***  ***We present key elements of study design at the very beginning of the Methods section.*** |
| Setting | 5 | Describe the setting, locations, and relevant dates, including periods of recruitment, exposure, follow-up, and data collection  ***Response:***  ***We provide this information in the first paragraph of the Methods section.*** |
| Participants | 6 | (*a*) *Cohort study*—Give the eligibility criteria, and the sources and methods of selection of participants. Describe methods of follow-up  ***Response:***  ***Again, this information is provided in the first paragraph of the Methods section.***  *Case-control study*—Give the eligibility criteria, and the sources and methods of case ascertainment and control selection. Give the rationale for the choice of cases and controls  *Cross-sectional study*—Give the eligibility criteria, and the sources and methods of selection of participants |
|  |  | (*b*) *Cohort study*—For matched studies, give matching criteria and number of exposed and unexposed  *Case-control study*—For matched studies, give matching criteria and the number of controls per case |
| Variables | 7 | Clearly define all outcomes, exposures, predictors, potential confounders, and effect modifiers. Give diagnostic criteria, if applicable  ***Response:***  ***We described the primary outcome of interest (mortality) and the primary exposure of interest (patient sex). We named potential confounders as listed in our Figure 2 (including age and time on dialysis among numerous other patient and treatment characteristics).*** |
| Data sources/ measurement | 8* | For each variable of interest, give sources of data and details of methods of assessment (measurement). Describe comparability of assessment methods if there is more than one group  ***Response:***  ***Mortality was collected during follow-up and sex was described at study entry.*** |
| Bias | 9 | Describe any efforts to address potential sources of bias  ***Response:***  ***We state that patients participating in the DOPPS are randomly selected from each participating dialysis facility.*** |
| Study size | 10 | Explain how the study size was arrived at  ***Response:***  ***We stated that we analyzed data [1] from 206,374 DOPPS census patients from the initial cross-section of patients in each study phase, i.e. all patients dialyzing in the DOPPS facilities at study start, having data on demographics and mortality, and [2] from 35,964 prevalent (on dialysis more than 90 days at study enrollment) DOPPS sample patients.*** |
| Quantitative variables | 11 | Explain how quantitative variables were handled in the analyses. If applicable, describe which groupings were chosen and why  ***Response:***  ***We indicate which variables are in each model and clearly indicate when continuous variables are categorized. The rationale for age group categories is described the methods section.*** |
| Statistical methods | 12 | (*a*) Describe all statistical methods, including those used to control for confounding |
|  |  | (*b*) Describe any methods used to examine subgroups and interactions |
|  |  | (*c*) Explain how missing data were addressed |
|  |  | (*d*) *Cohort study*—If applicable, explain how loss to follow-up was addressed  *Case-control study*—If applicable, explain how matching of cases and controls was addressed  *Cross-sectional study*—If applicable, describe analytical methods taking account of sampling strategy |
|  |  | (*e*) Describe any sensitivity analyses  ***Response:***  ***Our methods section describes the statistical methods, follow-up time calculations, sub-group analyses, and missing data. All sensitivity analyses are described and results provided in the supplemental tables and figures.*** |

Continued on next page

| Results | | |
| --- | --- | --- |
| Participants | 13* | (a) Report numbers of individuals at each stage of study—eg numbers potentially eligible, examined for eligibility, confirmed eligible, included in the study, completing follow-up, and analysed |
|  |  | (b) Give reasons for non-participation at each stage |
|  |  | (c) Consider use of a flow diagram  ***Response:***  ***The numbers of individuals are reported in the first two sentences of the manuscript.*** |
| Descriptive data | 14* | (a) Give characteristics of study participants (eg demographic, clinical, social) and information on exposures and potential confounders |
|  |  | (b) Indicate number of participants with missing data for each variable of interest |
|  |  | (c) *Cohort study*—Summarise follow-up time (eg, average and total amount)  ***Response:***  ***Characteristics of study participants are reported in Table 2. Median follow-up time and covariate missingness are described in the Methods section.*** |
| Outcome data | 15* | *Cohort study*—Report numbers of outcome events or summary measures over time  ***Response:***  ***The number of deaths is reported in the footnote for figure 2.*** |
|  |  | *Case-control study—*Report numbers in each exposure category, or summary measures of exposure |
|  |  | *Cross-sectional study—*Report numbers of outcome events or summary measures |
| Main results | 16 | (*a*) Give unadjusted estimates and, if applicable, confounder-adjusted estimates and their precision (eg, 95% confidence interval). Make clear which confounders were adjusted for and why they were included  ***Response:***  ***This was done.*** |
|  |  | (*b*) Report category boundaries when continuous variables were categorized  ***Response:***  ***Done.*** |
|  |  | (*c*) If relevant, consider translating estimates of relative risk into absolute risk for a meaningful time period  ***Response:***  ***This manuscript was particularly interested in the relative risk of mortality for males vs. females.*** |
| Other analyses | 17 | Report other analyses done—eg analyses of subgroups and interactions, and sensitivity analyses  ***Response:***  ***This was done-see in particular the supplementary materials.*** |
| Discussion | | |
| Key results | 18 | Summarise key results with reference to study objectives  ***Response:***  ***As stated in the beginning of the Discussion, we found [regarding objective (1) to assess hemodialysis prevalence among study participants, overall and by country], that fewer women than men were undergoing hemodialysis treatment in our sample, consistent with national hemodialysis registry data, despite higher proportions of women in the general population across all age groups. Regarding objective (2) to assess national differences in sex-dependent hemodialysis patient mortality, we found that the survival advantage that women have over men in the general population was markedly diminished in the DOPPS hemodialysis population. Regarding objective (3) to assess sex-dependent differences in hemodialysis characteristics, we found that there were substantial cross-sectional differences between men and women on hemodialysis. Regarding objective (4) to assess the presence of a sex interaction in the associations between hemodialysis characteristics and mortality, we found that certain hemodialysis characteristics showed a significant sex interaction with mortality and may become targets to improve outcomes.*** |
| Limitations | 19 | Discuss limitations of the study, taking into account sources of potential bias or imprecision. Discuss both direction and magnitude of any potential bias  ***Response:***  ***We acknowledged several limitations: namely, that “he presented analyses of adjusted mortality risk can only show associations, not causations, and can thus merely hint at the mechanisms that render mortality rates similar in men and women on hemodialysis.” Likewise, that “our descriptive findings of hemodialysis prevalence by gender cannot answer why the prevalence is higher for men than women, that, however, the large national differences we identified suggest that the reasons may go beyond biological ones.”*** |
| Interpretation | 20 | Give a cautious overall interpretation of results considering objectives, limitations, multiplicity of analyses, results from similar studies, and other relevant evidence  ***Response:***  ***We attempted to fulfil this requirement to the best of our knowledge.*** |
| Generalisability | 21 | Discuss the generalisability (external validity) of the study results  ***Response:***  ***We discussed that the prevalence and mortality data are probably valid because they were confirmed by registry data and in a previous analysis that had a different focus.*** |
| Other information | | |
| Funding | 22 | Give the source of funding and the role of the funders for the present study and, if applicable, for the original study on which the present article is based  ***Response:***  ***We provided our funding statement at the end of the text.*** |

*Give information separately for cases and controls in case-control studies and, if applicable, for exposed and unexposed groups in cohort and cross-sectional studies.

**Note:** An Explanation and Elaboration article discusses each checklist item and gives methodological background and published examples of transparent reporting. The STROBE checklist is best used in conjunction with this article (freely available on the Web sites of PLoS Medicine at http://www.plosmedicine.org/, Annals of Internal Medicine at http://www.annals.org/, and Epidemiology at http://www.epidem.com/). Information on the STROBE Initiative is available at www.strobe-statement.org.
